# Analysis of gene expansion and defense-related genes in Anacardiaceae family from an evolutionary aspect

**DOI:** 10.3389/fpls.2025.1638044

**Published:** 2025-07-16

**Authors:** Bing-Liang Fan, Lin-Hua Chen, Ling-Ling Chen

**Affiliations:** State Key Laboratory for Conservation and Utilization of Subtropical Agro-bioresources, College of Life Science and Technology, Guangxi University, Nanning, China

**Keywords:** Anacardiaceae, comparative genomics, phylogenomics, gene family expansion, defense-associated genes, transposable elements, WRKY transcription factors

## Abstract

**Introduction:**

The Anacardiaceae family, encompassing economically and ecologically significant genera such as *Rhus*, *Mangifera*, and *Pistacia*, exhibits substantial genomic diversity and adaptive complexity. However, comparative genomic analysis and evolutionary insights into defense-related mechanisms within this family remain underexplored.

**Methods and results:**

This study employed a comprehensive phylogenomic, synteny, and gene family analysis across six *Rhus* species and three additional Anacardiaceae plants (*Mangifera indica*, *Pistacia vera*, and *Anacardium occidentale*). Our findings revealed distinct evolutionary trajectories: *Mangifera*/*Anacardium* underwent lineage-specific whole-genome duplications (WGDs) with chromosomal rearrangements, while *Rhus*/*Pistacia* retained only the ancestral gamma duplication. *Pistacia*’s genome expanded via transposable elements (TEs), whereas *Rhus* conserved chromosomal synteny despite accumulating TE-mediated structural variations. Some defense-related gene families, including WRKY transcription factors and nucleotide-binding leucine-rich repeat (NLR) genes, displayed substantial expansions and stress-responsive expression patterns, with 31 WRKY genes significantly upregulated during aphid infestation. NLRs clustered on chromosomes 4/12 showed positive selection signatures. Long terminal repeat retrotransposons exhibited Pleistocene-era activation bursts, potentially linked to climatic adaptation.

**Discussion:**

This study unveils TE-mediated diversification as a key driver of Anacardiaceae evolution, complementing WGD-dependent strategies in tropical lineages. The identification of lineage-specific structural variations, dynamic TE activities, and clustered defense-related genes highlights adaptive trade-offs shaped by biotic stresses and the biogeographic history of Anacardiaceae species. This study lays the groundwork for leveraging these genomic resources to enhance stress resilience and adaptive potential in economically important Anacardiaceae crops.

## 1 Introduction

The family Anacardiaceae, first proposed and systematically classified in the late 18th to early 19th century, belongs to the order Sapindales within the eudicot clade Rosids (Müller-Wille and Reeds, 2007; Lindley, 2015). This family comprises over 80 genera and 900 species, predominantly distributed across tropical regions of Africa, Asia, and the Americas, with some species extending into subtropical and temperate zones (Dahlgren, 1982; Wannan, 2006). It is classified into five tribes: Anacardieae, Dobineeae, Rhoeae, Semecarpeae, and Spondiadeae (Pell, 2004). Members of the Anacardiaceae family are mainly woody plants, including trees, shrubs, and lianas, while herbaceous forms are rare (Baskin and Baskin, 2022). The family is distinguished by its remarkable ecological adaptability, diverse array of secondary metabolites, and substantial economic importance, making it a vital component of both natural ecosystems and human industries. Notable examples include tropical fruits such as the mango (*Mangifera indica*), cashew (*Anacardium occidentale*), and pistachio (*Pistacia vera*), which are globally cultivated for their edible fruits and seeds (Zeng et al., 2019; Wang et al., 2020c; Mango Genome Consortium et al., 2021). Additionally, several species yield high-quality timber that is resistant to decay, contributing to niche wood markets. The family also holds horticultural importance; for instance, *Cotinus coggygria* (smoke tree) is widely planted for its ornamental foliage (Matić et al., 2016). Medicinally, extracts from plants such as *Pistacia chinensis* (Chinese pistache) have been utilized in traditional remedies for their anti-inflammatory and antimicrobial properties (Paterniti et al., 2017). Beyond its horticultural and medicinal applications, *Toxicodendron vernicifluum* (Chinese lacquer tree) serves as a vital source of raw lacquer (Wang et al., 2020b). By tapping its trunk, this species yields a natural adhesive and coating material renowned for its remarkable properties, such as anticorrosion, antirust, nonoxidation, and resistance to acids, alcohol, and high temperatures (Suzuki et al., 2007; Zhao et al., 2013; Suzuki et al., 2014). These qualities make it indispensable for both traditional craftsmanship and modern industrial applications. Furthermore, a unique feature of *Rhus* species is their intricate interaction with aphids, which induces the formation of Chinese gallnuts (Wu-bei-zi) (Chen et al., 2020; Wang et al., 2020a; Wei et al., 2022). These galls are exceptionally rich in tannins, predominantly gallotannins, and are extensively utilized in traditional Chinese medicine, the chemical industry, and as animal feed additives due to their potent bioactive properties (Zhang et al., 2014; Ren et al., 2021). The family produces a wide array of secondary metabolites, many of which underpin its economic and ecological roles. Key compounds include urushiols (allergenic phenols in lacquer trees) (Ma et al., 2012), anacardic acids (cashew-derived antimicrobial agents) (Dendena and Corsi, 2014), mangiferolic acids (bioactive triterpenoids in mango) (Ediriweera et al., 2017), and tannins (e.g., gallic acid and catechols in gallnuts) (Beretta et al., 2009). These metabolites contribute to defense mechanisms against herbivores and pathogens while offering industrial applications, such as tannins for leather processing and urushiols for producing durable lacquers (Kumanotani, 1995; Chen et al., 2018).

**Figure 1:**
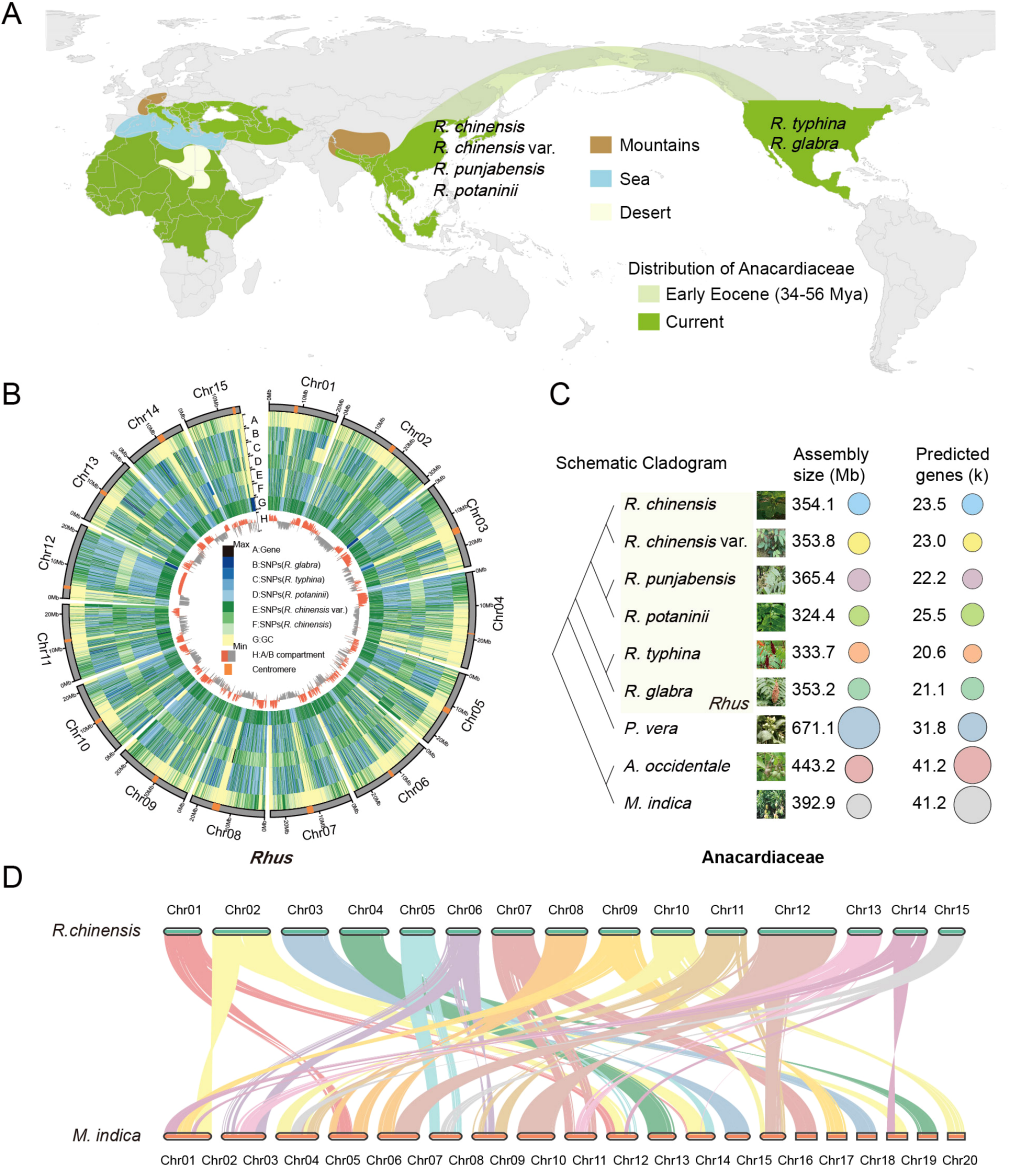
Origins and genomic features of Anacardiaceae. **(A)** Current and Eocene distribution of Anacardiaceae is shown in green and light green, respectively. **(B)** Circos plot of distribution of the genomic elements in five *Rhus* species, with *R. punjabensis* as the reference genome. (A–H) Concentric circles from outermost to innermost display protein-coding genes, SNP density in *R. glabra*, SNP density in *R. typhina*, SNP density in *R. potaninii*, SNP density in *R. chinensis* var. *roxburghii*, SNP density in *R. chinensis*, GC content and A/B compartment respectively. Chromosomes with centromeres are highlighted in orange. **(C)** The assembled evolutionary relationships, genome sizes, and annotated gene counts of nine Anacardiaceae genomes are presented in this study. **(D)** Synteny and micro-synteny among *R. chinensis* and *M. indica*.

Anacardiaceae exhibits specialized adaptations for pollination and seed dispersal. Floral nectaries attract insect pollinators, while fruits or seeds facilitate dispersal by insects, birds, and mammals (Herrera et al., 2018). Fossil evidence suggests an ancient origin, with genera such as *Pistacia* and *Cotinus* identified in Mediterranean Tertiary deposits (Palamarev, 1989; Marino et al., 2018), and *Toxicodendron* (lacquer tree) and *Rhus* fossils documented across Europe, North America, and Northeast Asia (Andreánszky, 1959; Wolfe, 1966; Yi et al., 2004; Jiang et al., 2019). This paleobotanical record highlights the family’s long-term evolutionary success and adaptability to diverse climates. The evolutionary history of Anacardiaceae is deeply intertwined with the Boreotropical flora that dominated the Northern Hemisphere during much of the Tertiary period, particularly from the Eocene to the middle Miocene (Wolfe, 1975). This tropical flora extended across North America, Europe, and into Asia. During the early Tertiary, the western part of North America and East Asia emerged as hotspots for the evolution and diversification of Tertiary relict plants, which later migrated to other parts of the Northern Hemisphere (Xiang, 2001). Botanists have long noted the significant floristic similarities across the Northern Hemisphere, dating back to observations made in the eighteenth century (Gray, 1857; Boufford and Spongberg, 1983; Wen, 1999). Among these, the well-known disjunction between East Asia and eastern North America is particularly striking, often referred to as the East Asia–eastern North America floristic disjunction. Climate deterioration following the late Eocene disrupted the Boreotropical belt, forcing thermophilic plants toward equatorial refugia. Southeastern Asia became a major sanctuary due to its continuous land connection to tropical zones (Morley, 2007), while Central/South America hosted fewer survivors (Burnham and Graham, 1999; Montes et al., 2015). Europe retained minimal Boreotropical elements due to Mediterranean barriers, Alpine uplift, and Sahara expansion (Tiffney, 1985; Morley, 2001). Within Anacardiaceae, the genus *Rhus* exemplifies this biogeographic narrative (**Figure 1A**). The oldest *Rhus* fossils from western North America (Early Eocene) and continuous records through Pliocene confirm North America as its origin center (Wilf, 2000; Yi et al., 2004). Dual eastward migrations occurred via Beringian and North Atlantic routes during the Eocene thermal maximum, when Boreotropical flora blanketed northern latitudes. Late Eocene *Rhus* fossils in Alaska corroborate Bering Land Bridge (BLB) dispersal, while late Miocene specimens resembling North American *R. glabra* and *R. typhina* in Hungary suggest North Atlantic Land Bridge (NALB) migrations (Andreánszky, 1959; Wolfe, 1966). Toxicodendron presents a complementary case of Boreotropical dynamics. Originating in the New World during late Eocene, it diverged into subtropical-temperate and tropical lineages before migrating to East Asia via NALB routes during Oligocene-early Miocene (Jiang et al., 2019). Most species spread from North America to East Asia, where they underwent adaptive radiation and increased species diversity, which is associated with East Asia’s climate and continuous land connection.

WRKY transcription factors constitute a major family of plant-specific regulators that play pivotal roles in modulating defense-related gene expression during biotic and abiotic stress responses (Wani et al., 2021; Javed and Gao, 2023). Acting as both positive and negative regulators of plant immunity, WRKYs participate in complex transcriptional networks that contribute to rapid transcriptional reprogramming and enhanced stress resilience (Eulgem and Somssich, 2007). Meanwhile, nucleotide-binding leucine-rich repeat (NLR) proteins are critical components of plant immune systems, mediating defense responses against a broad spectrum of pathogens through the recognition of specific pathogen-derived signals (Jacob et al., 2013; Yang et al., 2017). Despite their independent evolutionary origins in plants and animals, plant NLRs are often organized in genomic clusters, facilitating coordinated immune activation and contributing to both race-specific and broad-spectrum disease resistance (Wersch and Li, 2019). Furthermore, plant hormones such as salicylic acid (SA), jasmonic acid (JA), abscisic acid (ABA), and ethylene orchestrate intricate signaling networks and crosstalk mechanisms, fine-tuning immune responses, balancing defense and growth, and optimizing resource allocation under stress conditions (Vos et al., 2013; Verma et al., 2016; Berens et al., 2017). Collectively, these regulatory components underpin the sophisticated defense strategies plants employ to mitigate environmental challenges. With its rich phytochemical diversity, ecological versatility, and multifaceted human uses, Anacardiaceae represents a vital plant family bridging natural biodiversity and socioeconomic development. Despite its importance, evolutionary and adaptive studies within the family have historically been constrained by limited genomic resources, with prior phylogenetic reconstructions relying predominantly on chloroplast genomes or transcriptome-level data. To address these gaps, this study leverages chromosome-level genomes of six *Rhus* species assembled in our laboratory, combined with publicly available genomic data from globally cultivated species (*M. indica*, *P. vera*, and *A. occidentale*), to establish a robust phylogenomic framework. This approach not only resolves long-standing ambiguities in the family’s evolutionary relationships but also provides the first comparative genomic perspective on lineage-specific gene family expansions, particularly those linked to environmental adaptation and chemical defense. Furthermore, through bioinformatic analyses, we systematically unravel the structural diversity, copy-number variation, and selection pressures acting on defense-related genes (e.g., WRKY transcription factors, NLR genes, and plant hormone-related genes). By integrating evolutionary genomics with functional annotation, this work advances our understanding of how genomic innovation has driven the diversification and ecological dominance of Anacardiaceae across tropical to temperate ecosystems. Ultimately, these findings offer a foundation for targeted breeding of high-value species, sustainable utilization of bioactive compounds, and conservation strategies tailored to this economically and ecologically pivotal plant family.

## 2 Materials and methods

### 2.1 Data sources

The genomic and annotation data utilized in this study were obtained from two sources. First, six *Rhus* genomes assembled by our laboratory were included, achieving gold-standard reference genome quality to ensure the reliability and completeness of this research. Second, publicly available genomes of three additional Anacardiaceae species *Mangifera indica*, NCBI accession GCA_011075055.1 (Wang et al., 2020c), *Pistacia vera*, GCA_008641045.1 (Zeng et al., 2019), and *Anacardium occidentale* (Phytozome13, https://phytozome-next.jgi.doe.gov) were incorporated to broaden the phylogenetic scope (Goodstein et al., 2011). To provide an evolutionary framework for comparative analyses, the *Arabidopsis thaliana* genome (TAIR version 10) was included as an outgroup (Lamesch et al., 2011). This integrated dataset enabled a comprehensive investigation of genomic diversity and evolutionary dynamics within Anacardiaceae (**Supplementary Table S1**).

### 2.2 Synteny and whole-genome duplication analysis

Genome collinearity was investigated through JCVI (v1.2.7) using protein-based reciprocal best-hit alignments to detect conserved syntenic blocks (Tang et al., 2008). Putative orthologous relationships were established through pairwise genome comparisons, with aligned regions representing ancestral genomic linkages. Whole-genome duplication (WGD) chronology and speciation divergence times were resolved via WGDI (v0.6.1) analysis (Sun et al., 2022), incorporating calculation of nonsynonymous (Ka) and synonymous (Ks) substitution rates through the NG86 method (Nei and Gojobori, 1986). This integrative approach enabled temporal reconstruction of lineage-specific WGD events and interspecies divergence patterns.

### 2.3 Long terminal repeat retrotransposon characterization

Full-length long terminal repeat retrotransposons (LTR-RTs) were identified through complementary *de novo* prediction using LTRharvest (v1.5.10) (Ellinghaus et al., 2008) and LTR_FINDER_parallel (v1.1) (Ou and Jiang, 2019). Consensus predictions were integrated and refined through LTR_retriever (v2.9.0) (Ou and Jiang, 2018), with LTR insertion timing calculated using a lineage-specific substitution rate of 7×10^-9^ (Ossowski et al., 2010).

### 2.4 Variant detection and annotation

Genome-wide SNP identification was performed through MUMmer (v4.0.0beta2) alignment using stringent filtering parameters (-r -q -l 1000) to ensure reciprocal best matches (Marçais et al., 2018). The show-snps utility was implemented with -ClrTH thresholds to detect single nucleotide variants and small indels (< 100 bp). Functional impacts of identified polymorphisms were annotated using SnpEff (v5.0e) through comprehensive variant effect prediction (Cingolani et al., 2012). Visualization of the interchromosomal distribution of structural variations in *Rhus* species using SyRI (Goel et al., 2019).

### 2.5 Phylogenomic analysis

To investigate the evolutionary relationships within Anacardiaceae, we performed phylogenetic reconstruction incorporating six *Rhus* species from this study along with three additional Anacardiaceae taxa (*P. vera*, *M. indica*, *A. occidentale*) and the outgroup species *A. thaliana*. Single-copy ortholog identification was achieved through OrthoFinder (v2.5.4) analysis (Emms and Kelly, 2019), employing the longest transcript isoforms from ten plant species as input data. Multiple sequence alignment of conserved orthologous sequences was executed using MUSCLE (v5.1.linux64) (Edgar, 2022), followed by identification of phylogenetically informative regions through Gblocks (v0.91b) for site selection (Castresana, 2000).

Optimal substitution model selection via Prottest (v3.4.2) identified Blosum62+I+G+F as the most appropriate amino acid substitution matrix (Darriba et al., 2011). Maximum likelihood phylogenetic inference was subsequently conducted using RAxML (v8.2.12) with 200 bootstrap replicates to assess nodal support (Stamatakis, 2014). Temporal calibration of divergence events was implemented in MCMCTREE (PAML v4.9j) (Yang, 2007), incorporating a key fossil-derived calibration point (*Rhus*-*P. vera* split at ~48 mya) from TimeTree and prior paleobotanical studies (Moran, 1989; Kumar et al., 2017).

Gene family dynamics were analyzed through CAFE (v5.0) (Mendes et al., 2021), leveraging OrthoFinder-derived gene clusters to detect significant expansions/contractions (conditional *P* < 0.05 threshold). Functional enrichment analysis of dynamically evolving gene families was conducted using the R package clusterProfiler by performing Gene Ontology (GO) term and Kyoto Encyclopedia of Genes and Genomes (KEGG) pathway enrichment analyses (Ogata et al., 1999; Ashburner et al., 2000; Yu, 2024). KEGG pathway annotations were retrieved from the official database (http://www.kegg.jp/kegg/pathway.html).

### 2.6 Defense-related gene annotation and functional classification

To systematically identify and characterize defense-associated genes, we implemented an integrated annotation framework combining structural domain verification and functional ontology analysis. The WRKY transcription factors were rigorously identified by detecting the presence of the PF03106 WRKY domain through Pfam profiles. Specifically, the Pfam database integrated within the InterPro platform (https://www.ebi.ac.uk/interpro/) was queried using Hidden Markov Model (HMM) profiles with a stringent E-value cutoff of 1e-5 to ensure high-confidence annotation of these conserved protein domains (Blum et al., 2024). NLR-type resistance genes were classified based on the presence of characteristic nucleotide-binding domains (PF00931/NB-ARC, PF01582/TIR, PF05659/RPW8) coupled with leucine-rich repeat motifs (PF00560/LRR_1, PF07725/LRR_8, PF13306/LRR_9, PF13855/LRR_6). Functional annotation was extended through GO enrichment analysis targeting seven phytohormone signaling pathways: auxin (GO:0009734), cytokinin (GO:0009736), abscisic acid (GO:0009738), brassinosteroid (GO:0009742), jasmonic acid (GO:0009867), ethylene-activated signaling pathway (GO:0009873), and strigolactone (GO:0010476) responses. Additionally, selective pressure analysis was performed using PAML to calculate the dN/dS ratios of defense-related genes relative to background genes.

### 2.7 Phylogenetic relationship, gene structure, and conserved motifs analysis

To investigate the evolutionary relationships of defense-associated genes in Anacardiaceae, multiple sequence alignment of WRKY proteins identified in *Rhus* and *Arabidopsis* was performed using MUSCLE (v5.1.linux64). A neighbor-joining phylogenetic tree was subsequently constructed with 1,000 bootstrap replicates in MEGA11 to assess node reliability (Koichiro et al., 2021). Conserved motifs were predicted using the MEME Suite (v5.5.7) with default parameters (Bailey et al., 2015), while functional domains were annotated via the NCBI Conserved Domain Database (CDD) (Marchler-Bauer et al., 2014). Gene structure features (coding sequences and untranslated regions) and chromosomal localization patterns were visualized using TBtools (v2.225) to integrate genomic architecture and positional context (Chen et al., 2023).

### 2.8 Cis-regulatory element identification and protein network prediction

We retrieved 2-kb upstream promoter regions of *R. chinensis* WRKY family genes using genome annotation data. Putative cis-regulatory elements were systematically characterized through computational analysis using PlantCARE (https://bioinformatics.psb.ugent.be/webtools/plantcare/html/), with particular emphasis on stress-responsive motifs. Core promoter elements, such as the TATA-box and CAAT-box, were excluded from subsequent analyses to specifically focus on defense-related regulatory sequences. Visualization of spatial element distribution was performed using TBtools with customized annotation parameters (Chen et al., 2023). For protein interaction network inference, a homology-based approach was implemented. All annotated *R. chinensis* protein sequences were compiled into a local BLAST database. WRKY family proteins served as query sequences for iterative BLAST searches with E-value < 1e-5 and sequence identity >= 30%. The network topology was constructed using Gephi (Bastian et al., 2009), in which nodes represent individual WRKY proteins and edges denote significant sequence homology, indicating potential functional associations.

### 2.9 Transcriptomic profiling and differential expression analysis

Following specimen acquisition, tissues were rapidly preserved through cryogenic freezing to maintain biomolecular stability. Nucleic acid isolation was performed employing commercial plant RNA extraction kits (Tiangen Biotech) with subsequent enzymatic purification using recombinant DNase I (Takara Bio) to eliminate genomic DNA interference. RNA integrity verification was conducted through nucleic acid electrophoresis utilizing 1% agarose matrices impregnated with fluorescent nucleic acid stain, supplemented by dual-platform validation of nucleic acid purity and concentration through spectrophotometric (NanoDrop 2000C) and microfluidic (Agilent Bioanalyzer 2100) quantification systems. For Illumina sequencing, poly-A enriched transcripts were converted into amplifiable cDNA libraries through reverse transcription employing ultra-high-fidelity cDNA synthesis reagents (NEBNext series, NEB). The resulting PCR products were purified using the AMPure XP system, and the library quality was assessed on the Agilent Bioanalyzer 2100 system.

To ensure data reliability, three biological replicates were included for each sample. Raw sequencing data underwent comprehensive quality evaluation through FastQC (v0.12.1) prior to pre-processing (Andrews, 2010). Adapter trimming and read quality enhancement were implemented via Trim Galore (v0.6.7) (Krueger et al., 2021), followed by alignment of processed reads to the reference genome using HISAT2 (v2.2.1) with standard parameters (Kim et al., 2015). Transcript assembly was performed through StringTie (v2.1.0) (Pertea et al., 2015), with subsequent quantification of gene expression levels expressed as fragments per kilobase of transcript per million fragments mapped (FPKM). Differential expression analysis was executed through DESeq2 (v1.38.3) (Love et al., 2014), employing a stringent significance threshold (*P* < 0.01) coupled with a |log2 fold change| ≥ 1 for candidate gene identification.

### 2.10 Statistical analysis

The statistical details of analysis applied in this paper are provided alongside in the results and methods section. Statistical analyses were performed in R 4.0.0 (R Core Team, 2014).

## 3 Results

### 3.1 Genomic features of Anacardiaceae species

The Anacardiaceae family, which includes economically and ecologically important genera such as *Rhus*, *Mangifera*, and *Pistacia*, exhibits substantial genomic diversity and evolutionary complexity. To explore these variations, we used *R. punjabensis* as the reference genome for SNP calling across five other *Rhus* species (*R. chinensis*, *R. chinensis* var. *roxburghii*, *R. typhina*, *R. potaninii*, and *R. glabra*). Analysis of the chromosomal distribution revealed distinct compartmentalization, with the B compartment concentrated in centromeric regions, characterized by higher transposon density, while the A compartment was primarily distributed along chromosome arms, enriched with gene-dense regions (**Figure 1B**). This spatial organization reflects a high-quality genome assembly, reinforcing the reliability of SNP identification and interspecies comparisons. To further expand our comparative framework, we included three economically important species from the Anacardiaceae family: *P. vera*, *M. indica*, and *A. occidentale*, providing a broader perspective on genomic evolution within the family. As shown in **Figure 1C**, genome sizes varied significantly, ranging from 324.4 Mb in *R. potaninii* to 671.1 Mb in *P. vera*, with the number of genes spanning 20.6 k to 41.3 k (**Supplementary Table S2**). Notably, when *P. vera* was excluded, genome sizes of the remaining species showed relatively minor differences, indicating that large-scale expansions may be lineage-specific rather than characteristic of the entire family. Synteny analysis between *R. chinensis* and *M. indica* (**Figure 1D**) revealed a clear 1:2 syntenic relationship, suggesting that *M. indica* underwent a WGD event followed by chromosomal rearrangements, resulting in its 20-chromosome configuration. In contrast, *Rhus* species displayed a conserved chromosomal structure without evidence of recent WGD, implying that their diversification was primarily driven by smaller-scale duplications, SNP accumulation, and transposable element activities. These findings highlight not only the high assembly quality of the *Rhus* genomes but also the distinct evolutionary trajectories among major Anacardiaceae lineages.

### 3.2 Divergent genomic evolution in Anacardiaceae

**Figure 2:**
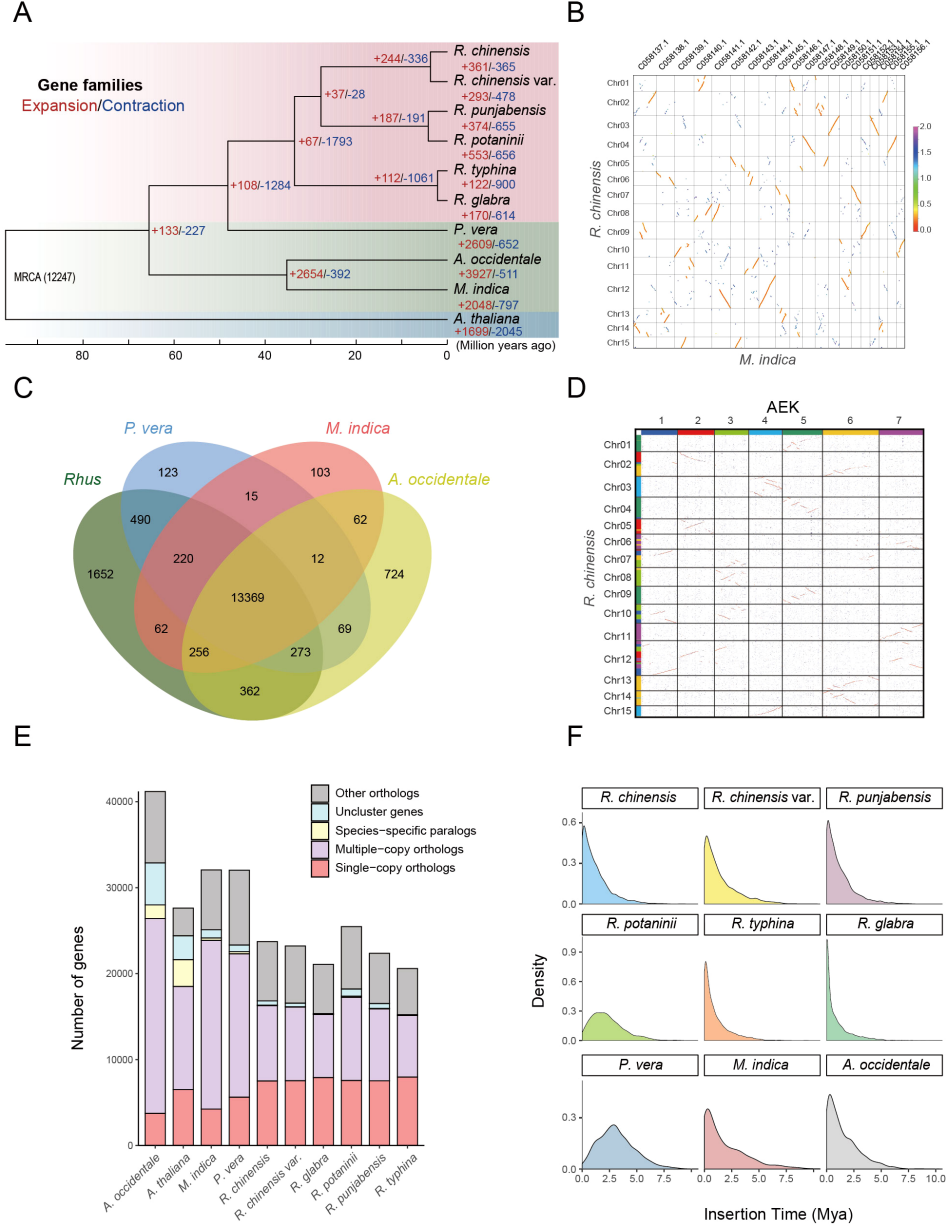
Comparative genomic and evolutionary analysis of Anacardiaceae species. **(A)** Phylogenetic tree of 9 Anacardiaceae species and *A. thaliana*, including their estimated divergence times based on orthologs from a single-gene family. Node supports were computed using 200 bootstrap replicates, with all nodes exhibiting 100% support. **(B)** Synteny blocks between *R. chinensis* and *M. indica*, with *M. indica* demonstrating a whole-genome duplication. **(C)** Clusters of orthologous and paralogous gene families in *Rhus* and three other Anacardiaceae species. **(D)** Synteny blocks between *R. chinensis* and the ancestral eudicot karyotype (AEK) indicate that *R. chinensis* has undergone a triplication event. **(E)** Clustering of gene families across different species. **(F)** Insertion times of long-terminal repeat retrotransposons (LTR-RTs) in 9 Anacardiaceae species.

Phylogenetic analysis based on whole-genome sequencing and orthologous gene families from nine representative Anacardiaceae species, with *A. thaliana* as an outgroup, enabled the reconstruction of evolutionary trajectories and divergence times. The resulting phylogenetic tree revealed that *Rhus* species form a distinct clade closely related to *Pistacia*. Molecular dating suggests that Anacardiaceae originated approximately 97 million years ago (Mya), with *Rhus* diverging from its common ancestor around 48 Mya (**Figure 2A**). This split likely reflects geographical isolation and subsequent ecological adaptation of *Rhus* across temperate and subtropical regions. Notably, significant gene family expansions were observed in *P. vera*, *M. indica*, and *A. occidentale*, potentially driven by WGD events and transposable element proliferation. Prior studies indicate that *M. indica* and *A. occidentale* share a common WGD event (Mango Genome Consortium et al., 2021), whereas synteny analysis between *R. chinensis* and *M. indica* demonstrated that *Rhus* did not undergo an independent WGD (**Figure 2B**). Comparative genomic analysis revealed that *Rhus* shares 13,369 gene families with three other Anacardiaceae species while possessing 1,652 unique gene families (**Figure 2C**). Further syntenic analysis with the ancestral eudicot karyotype (AEK) confirmed that *Rhus* experienced only the gamma duplication event shared among eudicots, without any lineage-specific WGD (**Figure 2D**). In total, 23,530 genes from *R. chinensis* were clustered into 16,411 gene families, of which 81.5% were conserved across *Rhus* and three other Anacardiaceae species, while 10.0% represented *Rhus*-specific families (**Figures 2C, E**). These unique gene families may contribute to the species-specific traits and ecological adaptability of *Rhus*.

### 3.3 Structural variations and retrotransposon dynamics in Anacardiaceae genomes

We conducted a comprehensive comparative analysis of structural variations (SVs) across *Rhus* species using *R. chinensis* as the reference genome. The analysis identified substantial differences in the number and types of genetic variations, including single nucleotide polymorphisms (SNPs), insertions/deletions (InDels), and SVs such as presence-absence variations (PAVs), inversions, and translocations. Among these species, *R. chinensis* var. *roxburghii* exhibited the highest number of SVs, with a total of 6,099 events, followed closely by *R. glabra* (5,355) and *R. typhina* (5,218). In terms of specific variation types, *R. potaninii* and *R. glabra* displayed the greatest number of deletions, with 143,237 and 143,254 events, respectively. Notably, the number of insertions was also prominent in *R. potaninii* (132,100), while *R. punjabensis* demonstrated the highest count of translocations (1,131). Comparative analysis illustrated clear lineage-specific patterns of SV distribution, as reflected in the contrasting SV profiles between *R. chinensis* var. *roxburghii* and *R. chinensis* (**Supplementary Figure S1**), *R. punjabensis* and *R. potaninii* (**Supplementary Figure S2**), and *R. typhina* and *R. glabra* (**Supplementary Figure S3**). These variations may underlie phenotypic divergence, ecological adaptation, and evolutionary differentiation among Anacardiaceae species. The identification of such structural variations provides valuable insights into the genomic mechanisms contributing to species-specific traits and adaptive capacities within Anacardiaceae.

Furthermore, transposable elements (TEs) have played a pivotal role in shaping genome architecture within Anacardiaceae. In contrast to *M. indica*, *P. vera* does not exhibit lineage-specific genome duplications, although it shares the ancient gamma duplication event common to eudicots. Instead, its larger genome size is primarily driven by substantial TE expansion, with TEs occupying a remarkable 70.7% of its genome and LTR-RTs accounting for 46.75%, rather than typical WGD observed in other species (Zeng et al., 2019). The insertion time distribution analysis of LTR-RTs in various Anacardiaceae species reveals multiple waves of transposition, potentially triggered by environmental shifts that facilitated adaptive evolution (**Figure 2F**). In *Rhus*, the insertion time analysis of intact LTR-RTs demonstrated that *R. chinensis* and *R. chinensis* var. *roxburghii* exhibit similar insertion patterns (**Supplementary Figure S4**), with recent transposition bursts observed in both lineages. Likewise, *R. typhina* and *R. glabra* showed comparable insertion dynamics (**Supplementary Figure S5**). In contrast, *R. punjabensis* and *R. potaninii* displayed distinct LTR insertion timelines, with *R. potaninii* experiencing a transposition burst approximately two million years ago, while *R. punjabensis* showed more recent LTR insertions around one million years ago (**Supplementary Figure S6**). The chromosomal distribution of retrotransposons suggests a potential role in enhancing genomic plasticity and adaptive capacity.

### 3.4 Gene family expansion and functional enrichment analysis in Anacardiaceae

**Figure 3:**
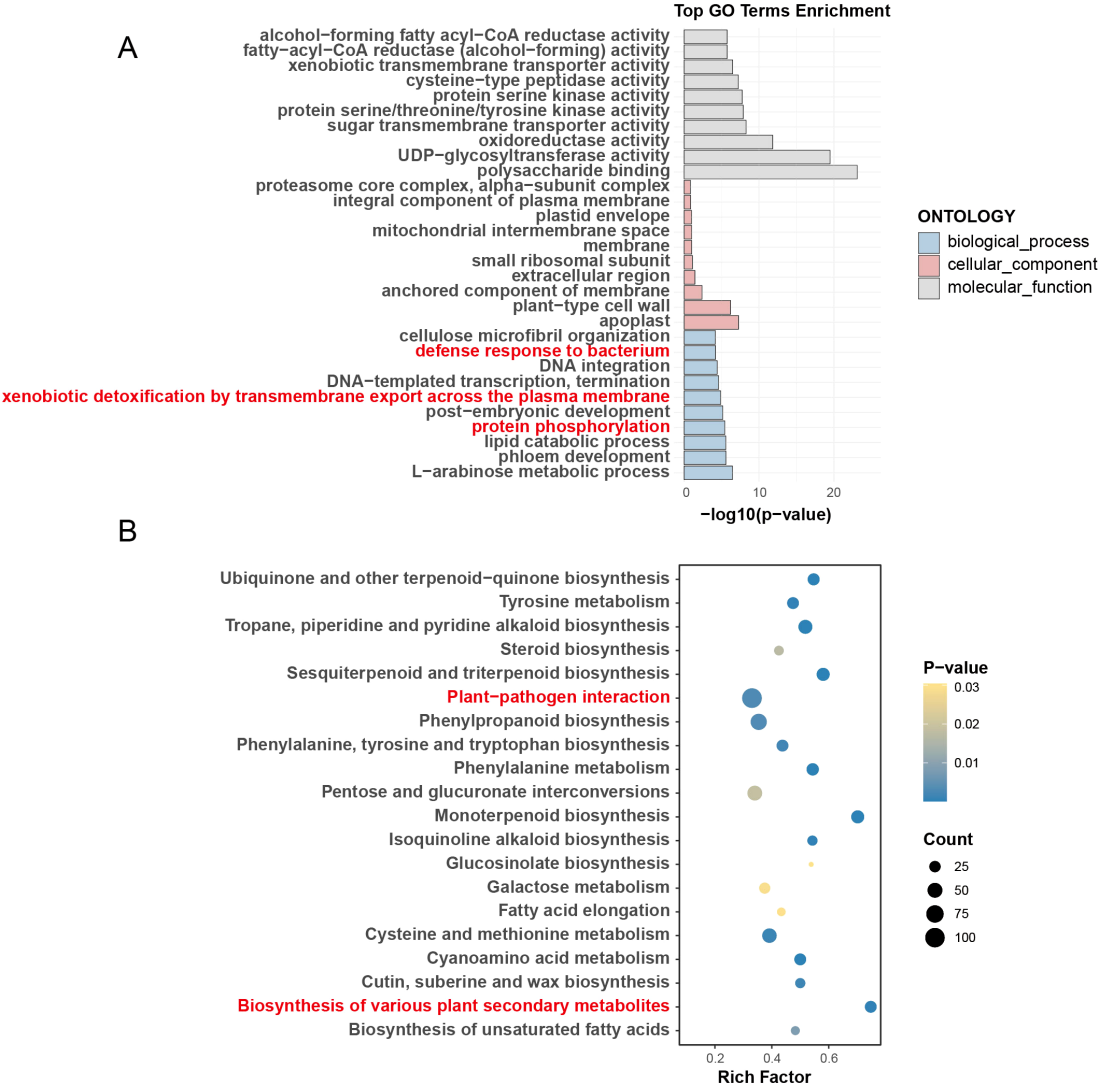
Functional enrichment analysis of expanded gene families in *P. vera*. **(A)** Gene Ontology (GO) enrichment and **(B)** Kyoto Encyclopedia of Genes and Genomes (KEGG) pathway enrichment.

Gene family expansion, a critical driver of adaptive evolution (Guo, 2013), was analyzed using the CAFE program across nine Anacardiaceae species. Notably, *P. vera*, *M. indica*, and *A. occidentale* exhibited significant expansions of 2,609, 2,048, and 3,927 gene families, respectively, with expansions notably outpacing contractions, suggesting that these species undergo adaptive evolution primarily through the expansion of specific gene families. Functional enrichment analyses revealed defense-related pathways as central to these expansions. For *P. vera* (**Figure 3**; **Supplementary Tables S3**, **S4**), GO terms such as “defense response to bacterium,” “xenobiotic detoxification by transmembrane export across the plasma membrane,” and “protein phosphorylation” were prominently enriched (**Figure 3A**). Correspondingly, KEGG pathways included “plant−pathogen interaction” and “biosynthesis of various plant secondary metabolites” (**Figure 3B**), highlighting their involvement in redox regulation, phytohormone-mediated defense signaling, and defense-associated metabolic processes. In Anacardiaceae, enriched GO terms included “defense response to bacterium,” “defense response,” and “signal transduction,” reflecting defense-related biological processes (**Supplementary Figure S7**; **Supplementary Tables S5**, **S6**). Similarly, *M. indica* showed significant enrichment in “regulation of defense response to fungus” and “response to auxin,” suggesting that the expanded gene families are predominantly associated with defense-related functions (**Supplementary Figure S8**; **Supplementary Tables S7**, **S8**). For *A. occidentale*, KEGG pathways such as “Plant hormone signal transduction” were highlighted, indicating its involvement in hormone-mediated defense responses (**Supplementary Figure S9**; **Supplementary Tables S9**, **S10**).

### 3.5 Phylogenetic and structural analysis of WRKY transcription factors in *Rhus* species

**Figure 4:**
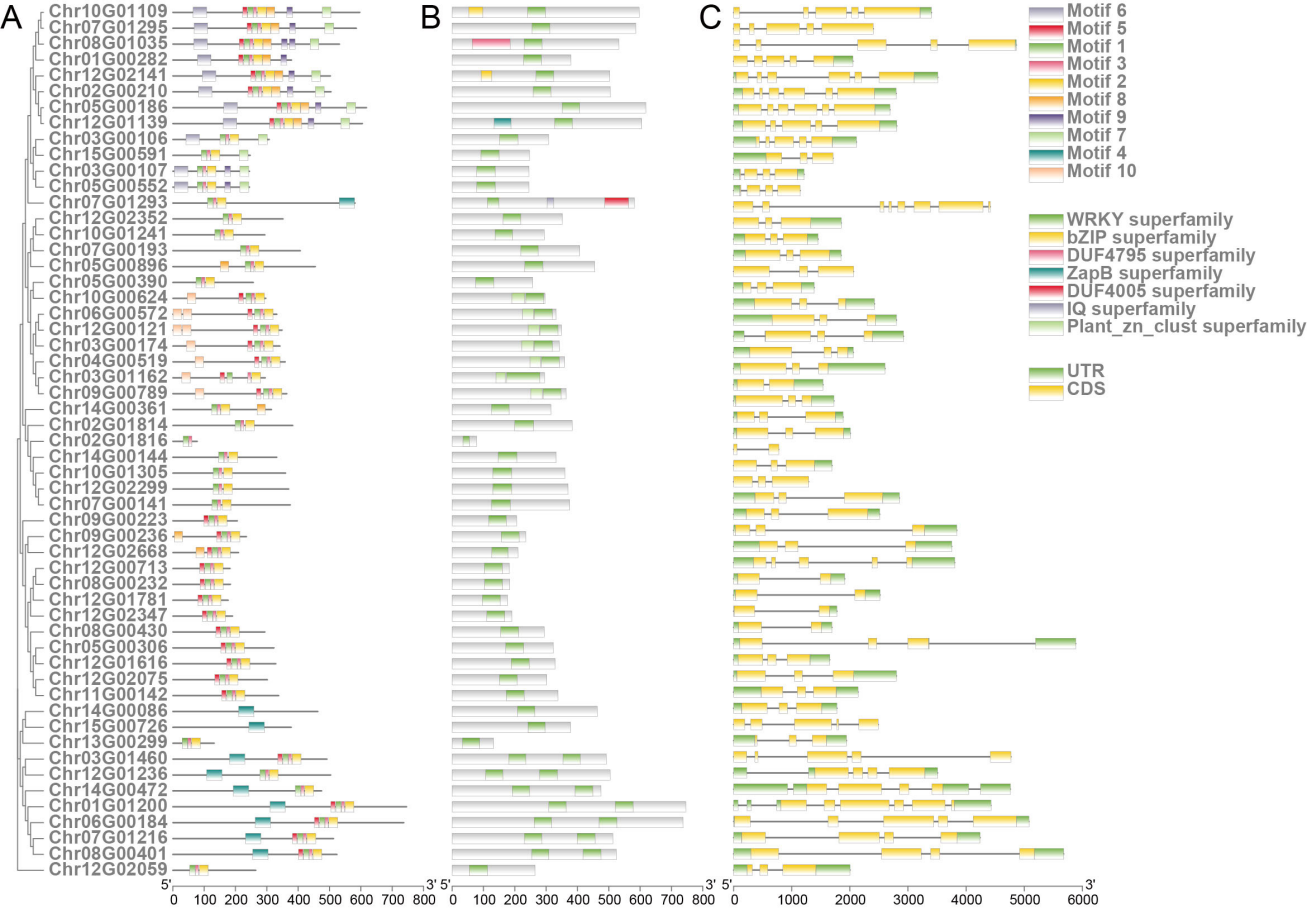
Structure of *R. chinensis* WRKY family members. **(A)** Phylogenetic relationships among WRKY family members (left) and distribution of conserved motifs in *R. chinensis* WRKY proteins (right). **(B)** Architecture of conserved domains in *R. chinensis* WRKY proteins. **(C)** Schematic structures of *R. chinensis* WRKY genes.

WRKY transcription factors (TFs) are pivotal regulators of plant defense mechanisms, orchestrating responses to biotic and abiotic stresses (Jiang et al., 2017). In *R. chinensis*, a comprehensive analysis was performed to investigate the phylogenetic relationships, conserved domains, and structural organization of WRKY TFs. Based on the conserved WRKY domain PF03106, a total of 55 WRKY genes were identified in *R. chinensis*. To elucidate their evolutionary relationships, 15 representative WRKY sequences from *A. thaliana* were retrieved from the TAIR database and aligned with the 55 *R. chinensis* WRKY sequences for phylogenetic analysis (**Supplementary Figure S10**; **Supplementary Table 11**). Structural analysis of the WRKY proteins revealed the presence of 10 conserved motifs distributed across the 55 members. These motifs exhibited distinct patterns among different WRKY subgroups, suggesting divergent evolutionary trajectories and specialized physiological roles (**Figure 4**; **Supplementary Table S12**). All *R. chinensis* WRKY proteins contained at least one WRKY superfamily domain. Additionally, several members harbored other conserved domains, such as plant-specific zinc finger motifs and bZIP superfamily elements, which may enhance transcriptional regulation under pathogen invasion and environmental challenges (**Figure 4**). Gene structure analysis further highlighted substantial variation in intron-exon arrangements among WRKY members. Some genes exhibited multiple introns, indicative of potential alternative splicing events that could contribute to functional diversity and regulatory complexity.

### 3.6 Cis-regulatory element profiling of WRKY gene promoters

**Figure 5:**
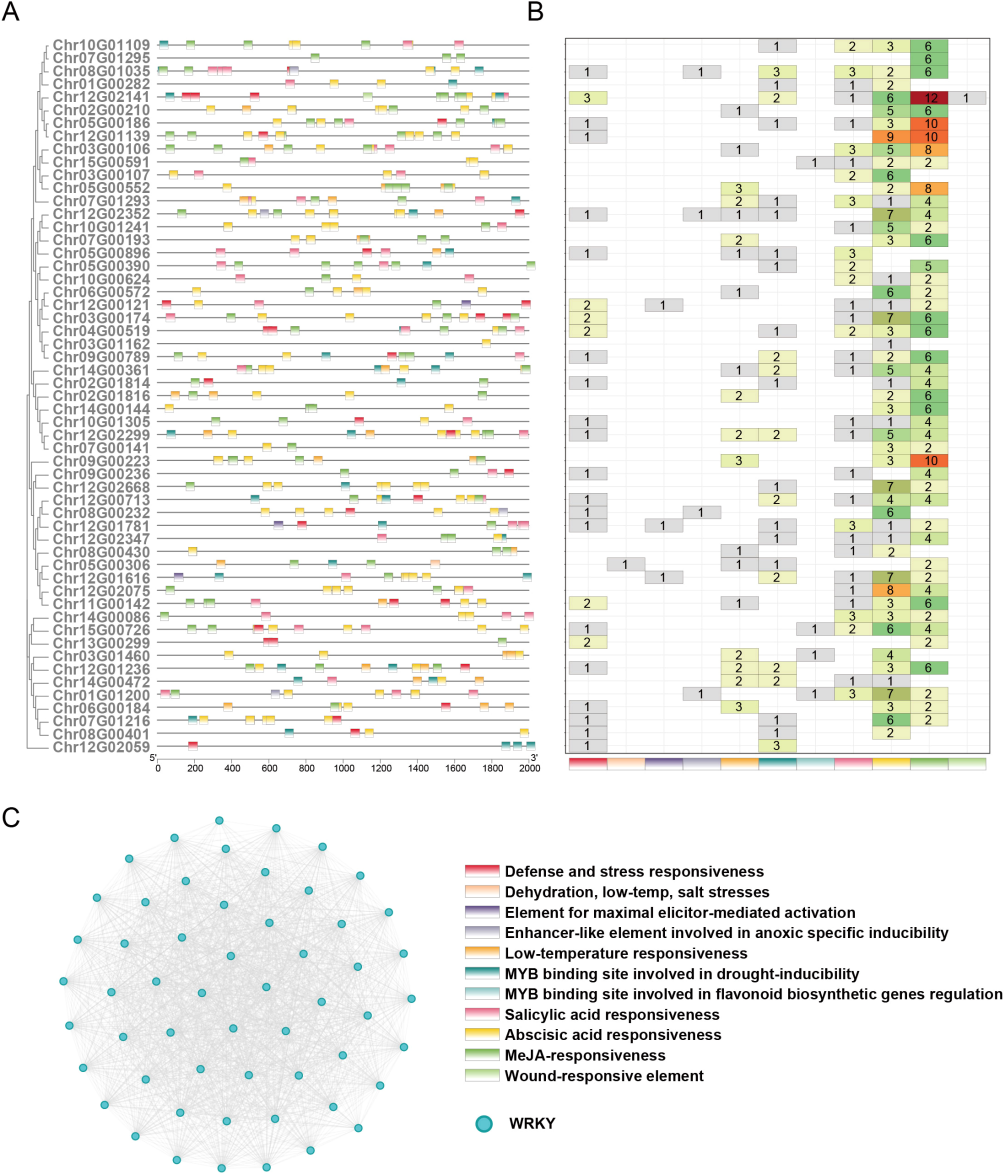
Defense-related cis-regulatory elements and protein interaction networks of WRKY gene family members. **(A)** Localization of 11 defense-related cis-acting elements within the 2-kb upstream promoter regions of WRKY genes. Distinct hues represent individual element types, and the horizontal scale denotes nucleotide positions relative to the transcription initiation site. **(B)** Frequency distribution of defense-related cis-element variants across WRKY gene family members. **(C)** The protein-protein interaction (PPI) network represents WRKY proteins as nodes, with edges (gray lines) denoting pairwise connections between proteins exhibiting >=30% sequence identity.

The functional diversity of genes is closely associated with the composition and abundance of cis-regulatory elements in their promoter regions. To investigate the potential regulatory mechanisms of WRKY transcription factors, we systematically analysed 2 kb upstream sequences of WRKY genes, with particular emphasis on cis-elements associated with abiotic stress adaptation, biotic stress responses, and defense metabolism regulation (**Figure 5A**). Our analysis revealed three predominant regulatory elements: MeJA-responsiveness (involved in jasmonate signaling), abscisic acid (ABA) responsiveness (central to drought and osmotic stress adaptation), and salicylic acid (SA) responsiveness (critical for systemic acquired resistance against pathogens), which emerged as the most prominently represented stress-related motifs (**Figure 5B**). These elements coordinate plant responses to environmental challenges through phytohormone-mediated signalling pathways. Furthermore, the promoter regions contained multiple specialized cis-regulatory modules, including elements governing low-temperature adaptation (e.g., dehydration, low-temperature, and salt stress-responsive motifs), hypoxia-inducible enhancer-like sequences, and MYB-binding sites regulating both drought-inducible gene expression and flavonoid biosynthesis. Notably, wound-responsive elements and pathogen defense motifs (e.g., elicitor-mediated activation elements) were selectively retained in specific WRKY promoters, implying specialized adaptation for localized stress recognition. The homology-based protein interaction network, constructed using sequence similarity thresholds, revealed a striking pattern of intra-family connectivity among WRKY proteins (**Figure 5C**). Nodes representing WRKY members exhibited dense clustering, with extensive edge formation signifying robust sequence homology-driven associations. Notably, no significant interactions were detected between WRKY proteins and non-WRKY proteins, suggesting functional modularity or selective evolutionary constraints that prioritize intramolecular cooperation within the WRKY family.

### 3.7 Distribution and evolutionary selection pressure of defense-associated genes

**Figure 6:**
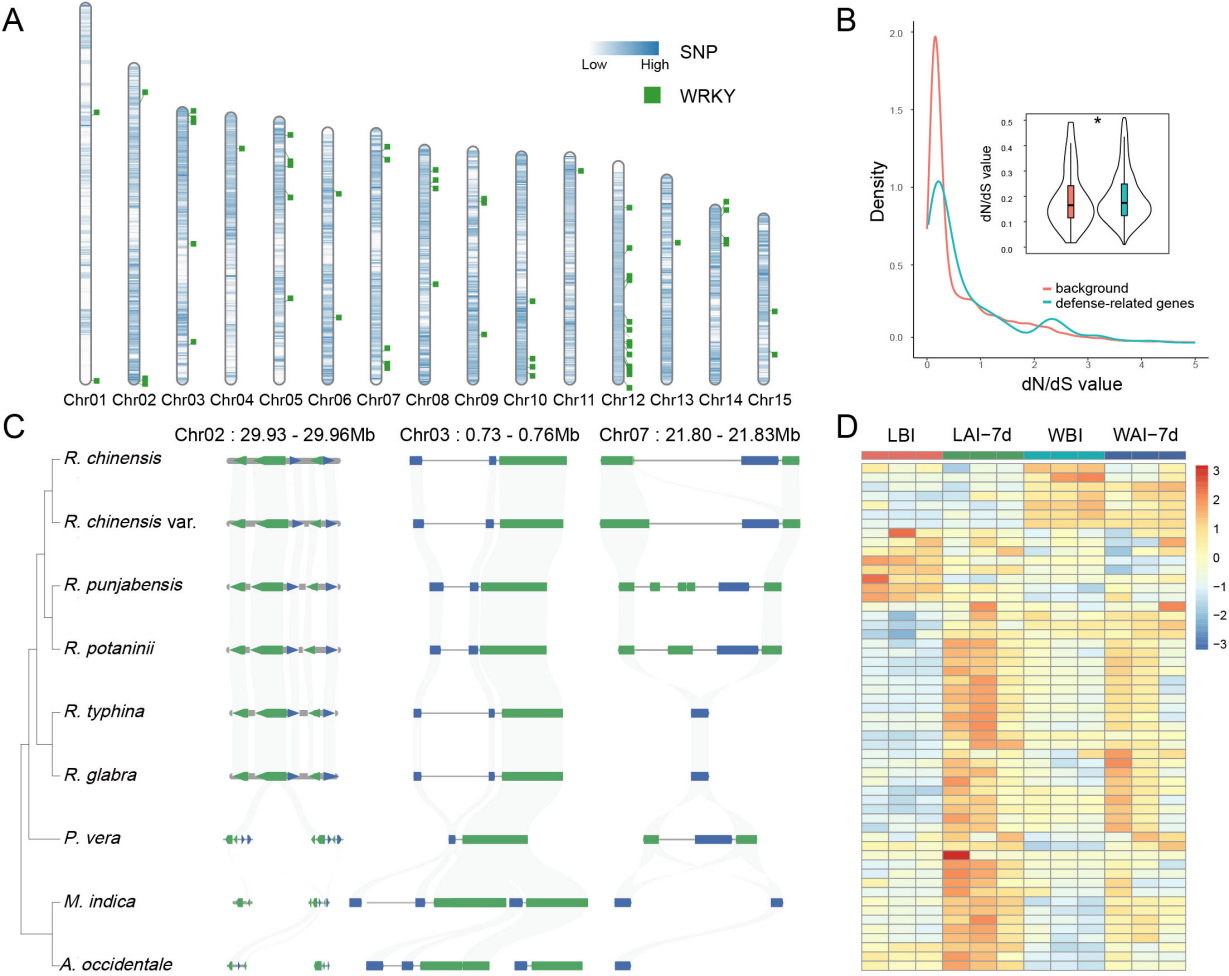
Genomic and expression features of WRKY-related defense genes in *R. chinensis*. **(A)** Chromosomal distribution of WRKY-related genes in the *R. chinensis* genome. Blue represents the density distribution of SNPs unique to Southeast Asian *Rhus* species. **(B)** Comparative assessment of evolutionary selection pressure (dN/dS ratios) between plant defense-associated genes and genome-wide background genes (*P < 0.05). **(C)** Synteny of defense-related WRKY genes across nine Anacardiaceae species. **(D)** Expression levels of WRKY-related genes in four conditions: leaf-wing before infection (WBI), leaf-wing after 7 days of infection (WAI-7d), leaf before infection (LBI), and leaf after 7 days of infection (LAI-7d).

Chromosomal mapping revealed that WRKY, NLR, and phytohormone-related genes are unevenly distributed across the chromosomes of *R. chinensis*. Notably, these genes exhibit clustered arrangements in specific chromosomal regions (**Figure 6A**; **Supplementary Figure S11**). Among them, NLR genes are particularly enriched on chromosomes 4 and 12 (**Supplementary Figure S12**), suggesting that these loci may play crucial roles in adaptive defense mechanisms. In *R. chinensis*, genome-wide identification and characterization of these defense-associated genes indicate their extensive presence across multiple chromosomes, with pronounced clustering at certain loci. This non-random distribution pattern implies potential hotspots for evolutionary adaptation, possibly driven by selective pressures to enhance pathogen defense. To further understand the evolutionary dynamics of these genes, we performed evolutionary analysis using the dN/dS ratio (nonsynonymous to synonymous substitution ratio). The analysis identified evidence of positive selection in these defense-related gene families (Student’s t-test, *P* < 0.05; **Figure 6B**), indicating that mutations in these loci are likely beneficial for improving defense responses against biotic stresses. These findings underscore the evolutionary importance of defense-associated genes in shaping the adaptive landscape of *Rhus* species.

### 3.8 Synteny and comparative analysis of defense-related genes across Anacardiaceae species

In this study, the synteny relationships of defense-related genes were analyzed among six *Rhus* species and three additional members of the Anacardiaceae family, namely *M. indica*, *P. vera*, and *A. occidentale*. High-resolution synteny maps revealed both the conservation and specificity of defense-related gene loci, with particular emphasis on regions associated with the WRKY gene family. The analysis primarily focused on syntenic clusters of WRKY-related genes, as illustrated in **Figure 6C**. Notably, conserved WRKY gene fragments were identified at the terminal or proximal regions of chromosomes 2, 3, and 7 across the six *Rhus* species, indicating strong syntenic relationships. Typically, these segments corresponded to a single copy in *P. vera*, whereas *M. indica* and *A. occidentale* maintained two copies, suggesting lineage-specific duplication events. Interestingly, certain deviations were observed. For example, on chromosome 7, the two North American *Rhus* species (*R. typhina* and *R. glabra*) exhibited specific loss of syntenic segments compared to the four East Asian *Rhus* species. Conversely, these syntenic regions were well-preserved in the more distantly related *P. vera*, indicating potential species-specific adaptive evolution.

### 3.9 Expression dynamics of defense-related genes under biotic stress

To validate the functional relevance of defense-associated genes identified in *Rhus* species, we investigated their transcriptional responses to infestation by gall-forming aphids, obligate parasites that induce tannin-rich galls (Wei et al., 2022). Samples were collected from four tissue-time point combinations: leaf-wing before infection (WBI), leaf-wing after 7 days of infection (WAI-7d), leaf before infection (LBI), and leaf after 7 days of infection (LAI-7d). RNA-seq analysis revealed that biotic stress significantly upregulated 31 WRKY transcription factor genes in leaves (LAI-7d vs. LBI; fold change >2; **Figure 6D**; **Supplementary Tables S13**, **S14**). Moreover, WRKY genes in leaf-wings (WAI-7d vs. WBI) exhibited a broad upregulation trend across the gene family, with expression levels increasing in the majority of members, though these changes did not reach statistical significance. Similarly, defense-related genes, including NLRs and phytohormone signaling components, exhibited pronounced expression shifts post-stress (**Supplementary Figure S13**). These results demonstrate that defense-related gene families under positive selection are functionally responsive to biotic stress.

## 4 Discussion

The Anacardiaceae family exemplifies contrasting evolutionary strategies shaped by chromosomal stability and TE-driven plasticity. While *M. indica* (2n = 40) and *A. occidentale* (2n = 42) underwent lineage-specific WGDs post-eudicot gamma duplication (Mango Genome Consortium et al., 2021; Savadi et al., 2022), *P. vera* (2n = 30) and *Rhus* species (2n = 30) retained ancestral karyotypes (Zeng et al., 2019; Ni et al., 2024), diverging via smaller-scale genomic changes. This aligns with prior hypotheses that TE-mediated expansion, rather than WGD, drives adaptation in taxa occupying stable niches (Bennetzen, 2005; Fedoroff, 2012; Lisch, 2013). Our phylogenomic analysis of nine Anacardiaceae species further resolves their Boreotropical origins, linking Miocene-Pliocene climatic shifts (~48 Mya) to the intercontinental disjunction of *Rhus* lineages. Notably, North American *R. typhina* and *R. glabra* exhibit morphological and genomic affinities to East Asian *R. potaninii* and *R. punjabensis* (Manchester and Judd, 2022), suggesting adaptive divergence during post-Eocene migrations. However, limited subspecies-level genomic data for *P. vera*, *M. indica*, and *A. occidentale* hinder precise reconstruction of their Tertiary biogeography, underscoring a need for expanded sampling.

Lineage-specific expansions of defense-related genes, including WRKY transcription factors and NLR receptors, highlight adaptive trade-offs within Anacardiaceae. In *Rhus*, chromosomal clustering of these genes (e.g., on chromosomes 4 and 12) mirrors defense “hotspots” observed in *Arabidopsis* and rice (Meyers et al., 2003; Singh et al., 2015), suggesting conserved mechanisms for co-regulation under stress. Synteny loss in North American *Rhus* WRKY loci contrasts with conservation in *P. vera*, implying geographic isolation reshaped regulatory architectures. Similarly, the TE-enriched genome of *P. vera*, which consists of 70.7% TEs, shows an expansion of NB-ARC and cytochrome P450 families (McHale et al., 2006; Zeng et al., 2019), potentially contributing to enhanced abiotic stress tolerance. This pattern is not observed in the WGD-dependent genomes of *M. indica* and *A. occidentale*. These findings challenge the primacy of WGD in plant diversification, instead implicating TE-mediated plasticity as a driver of ecological resilience. However, reliance on computational annotations limits functional validation, as syntenic deviations in *R. typhina* or *R. glabra* could reflect pseudogenization or regulatory divergence, necessitating transcriptomic profiling.

Our study repositions TEs as central players in Anacardiaceae evolution, particularly during Pleistocene climatic upheavals. Recent LTR retrotransposon bursts in *R. punjabensis* (~1 Mya) and *R. potaninii* (~2 Mya) coincide with major environmental shifts (Caves et al., 2015; Ding et al., 2017), suggesting that fluctuating climates may have stimulated TE mobilization. These findings align with the ‘genomic shock’ hypothesis, where environmental upheaval triggers TE activation to drive adaptive innovation (McClintock, 1984). Such TE dynamics mirror patterns observed in *Quercus* and *Pinus* (Plomion et al., 2018; Niu et al., 2022), where Pleistocene climatic oscillations contributed to genome expansion. In *P. vera*, rampant TE activity, which is absent in WGD-prone relatives, may have helped buffer against extinction during postglacial range fragmentation. Future studies should prioritize functional interrogation of TE impacts: CRISPR-edited *Rhus* lines could test whether LTR insertions near WRKY/NLR loci modulate stress responses. Additionally, comparative epigenomic analyses across Anacardiaceae may reveal how heterochromatin stability constrains or potentiates TE-driven adaptation (Slotkin and Martienssen, 2007), offering insights for breeding climate-resilient cultivars.

## Data Availability

The datasets presented in this study can be found in online repositories. The names of the repository/repositories and accession number(s) can be found in the article/**Supplementary Material**.
