# Supplementary material for: Analysis of gene expansion and defense-related genes in Anacardiaceae family from an evolutionary aspect
Source: Front Plant Sci. 2025 Jul 16;16:1638044. doi: 10.3389/fpls.2025.1638044 (PMC12307512; doi:10.3389/fpls.2025.1638044)
Supplement: Supplementary file 2 [file DataSheet1.docx]

Supplementary Material

# Supplementary Figures


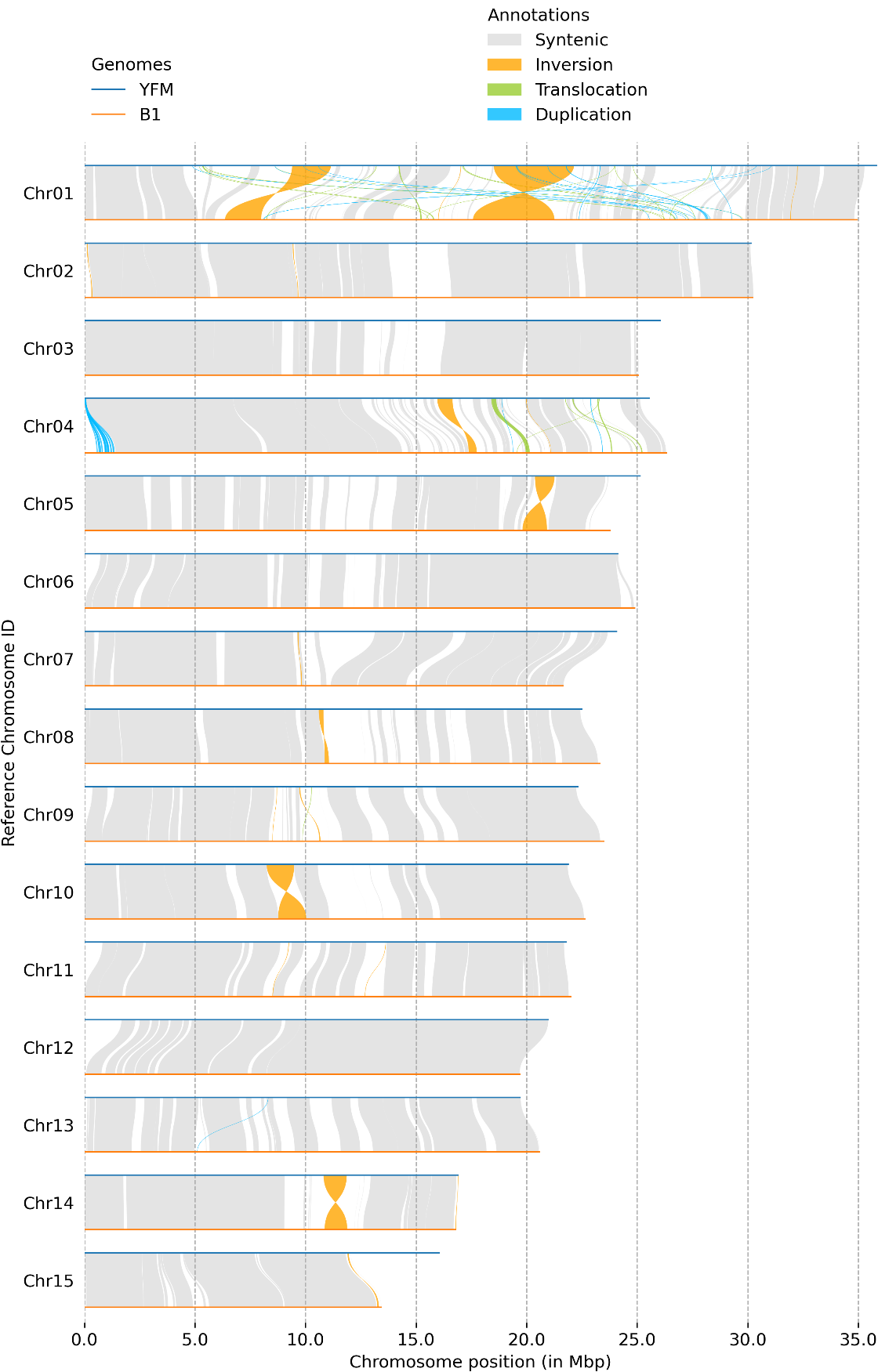


**Supplementary Figure 1.** **Legend for Structural Variations in Comparative Genomic Analysis.** The diagram depicts structural variations (SVs) between the *R. chinensis* (YFM) and *R. chinensis* var. *roxburghii* (B1), identified through comparative genomic analysis.

**
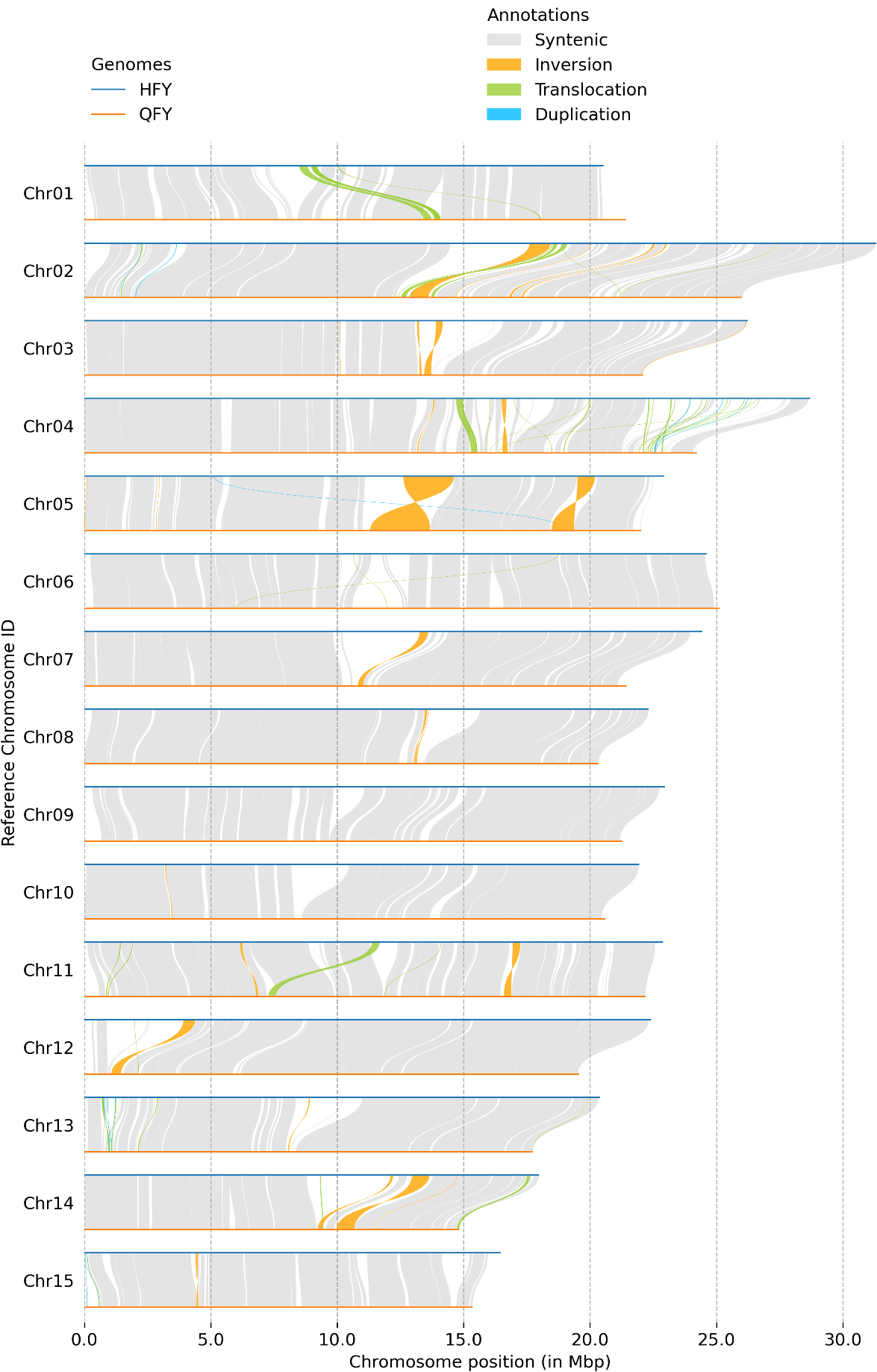
**

**Supplementary Figure 2.** **Legend for Structural Variations in Comparative Genomic Analysis.** The diagram depicts structural variations (SVs) between the *R. punjabensis* (HFY) and *R. potaninii* (QFY), identified through comparative genomic analysis.


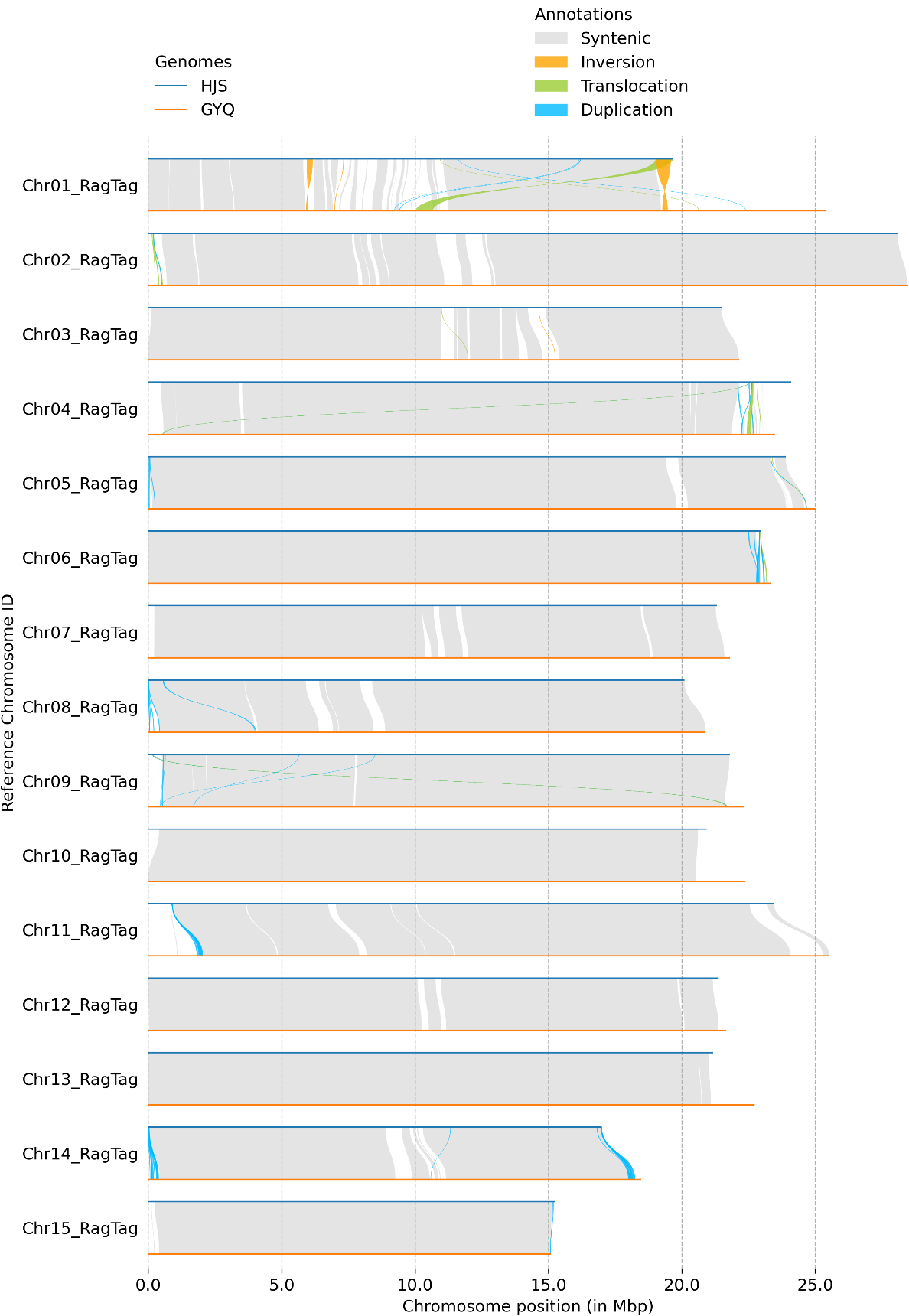


**Supplementary Figure 3.** **Legend for Structural Variations in Comparative Genomic Analysis.** The diagram depicts structural variations (SVs) between the *R. typhina* (HJS) and *R. glabra* (GYQ), identified through comparative genomic analysis.

**
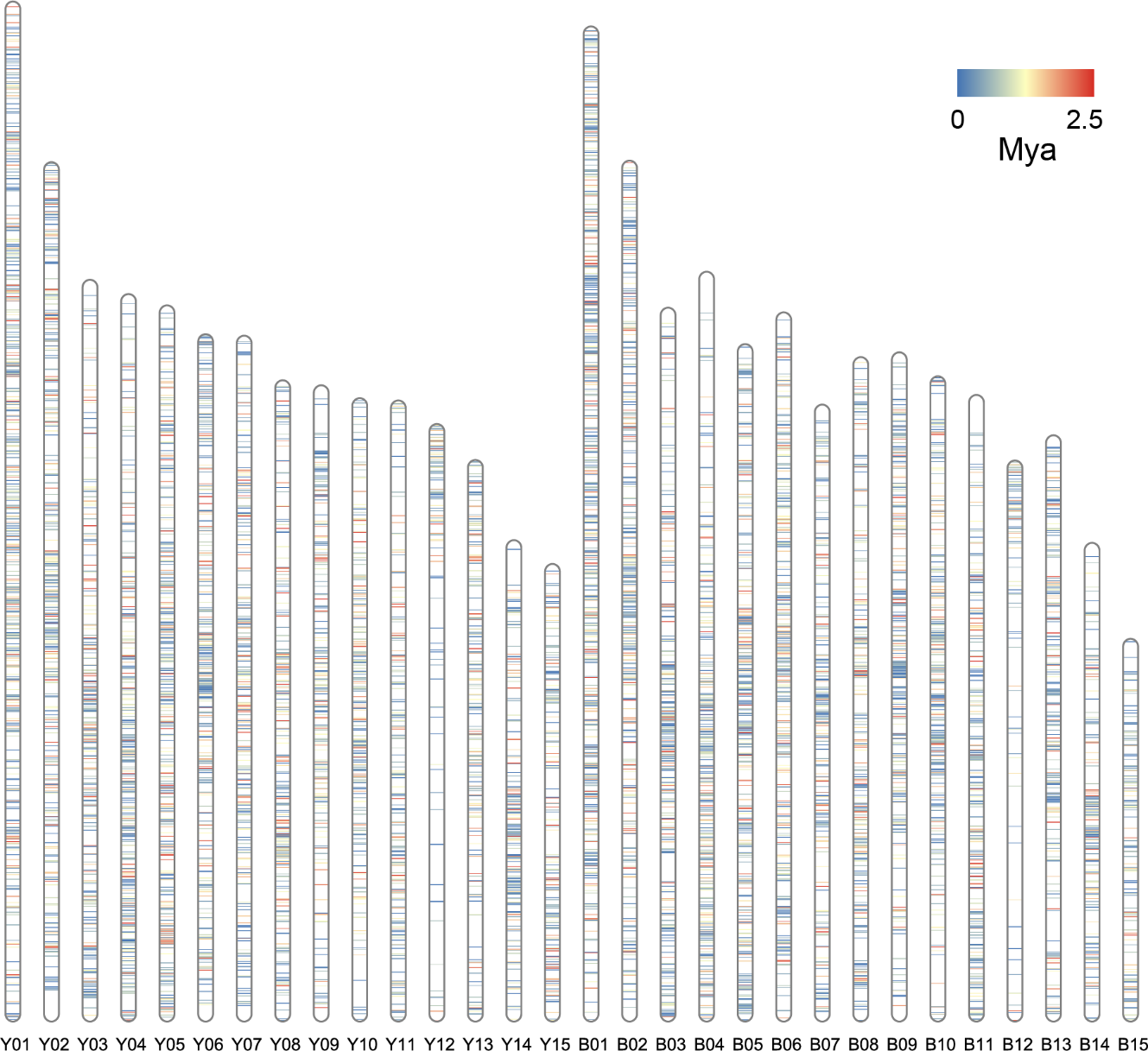
**

**Supplementary Figure 4.** Density distribution of long terminal repeat (LTR) insertion times and their respective chromosomal positions in *R. chinensis* (YFM) and *R. chinensis* var. *roxburghii* (B1).


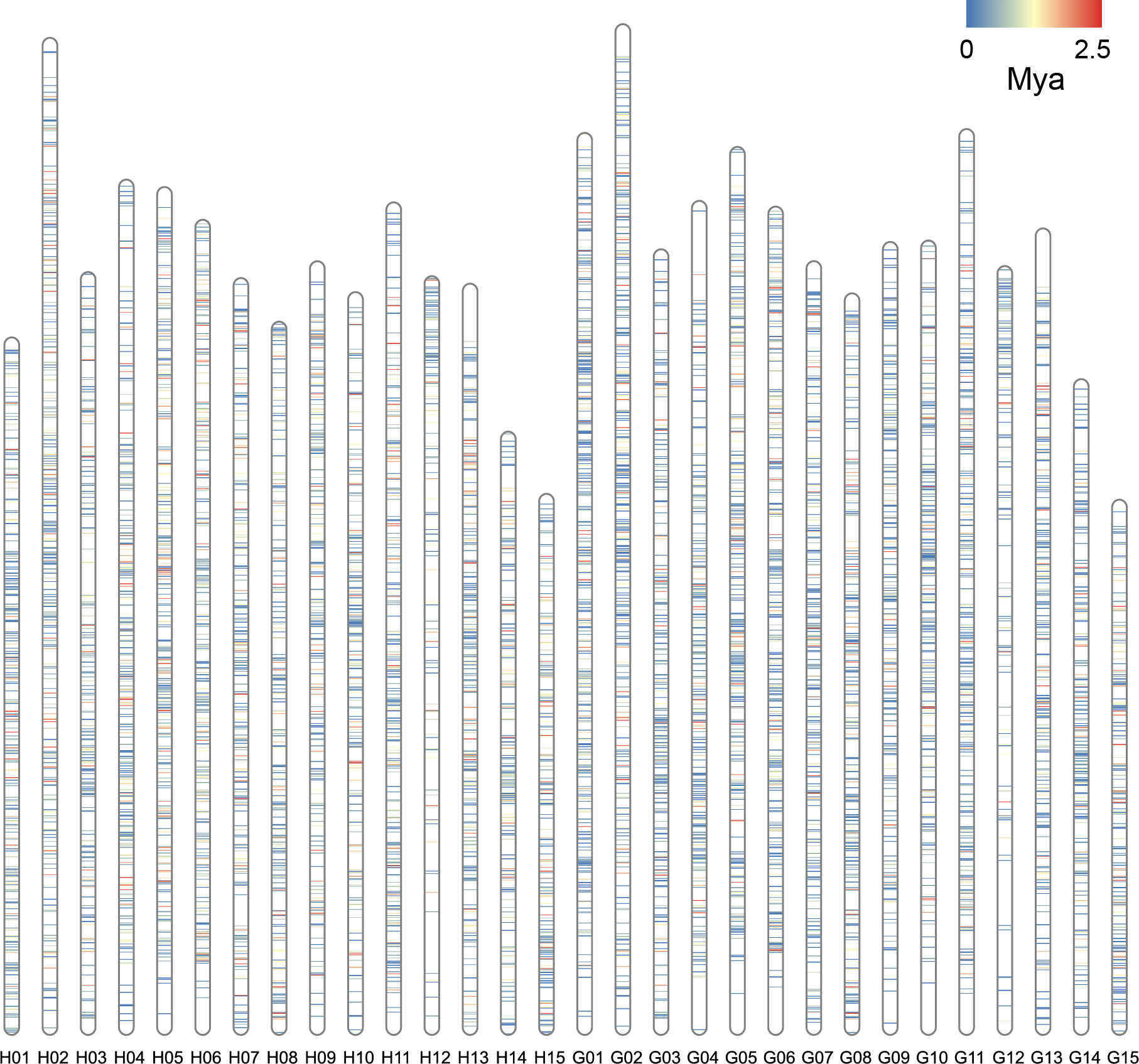


**Supplementary Figure 5.** Density distribution of long terminal repeat (LTR) insertion times and their respective chromosomal positions in *R. typhina* (HJS) and *R. glabra* (GYQ).


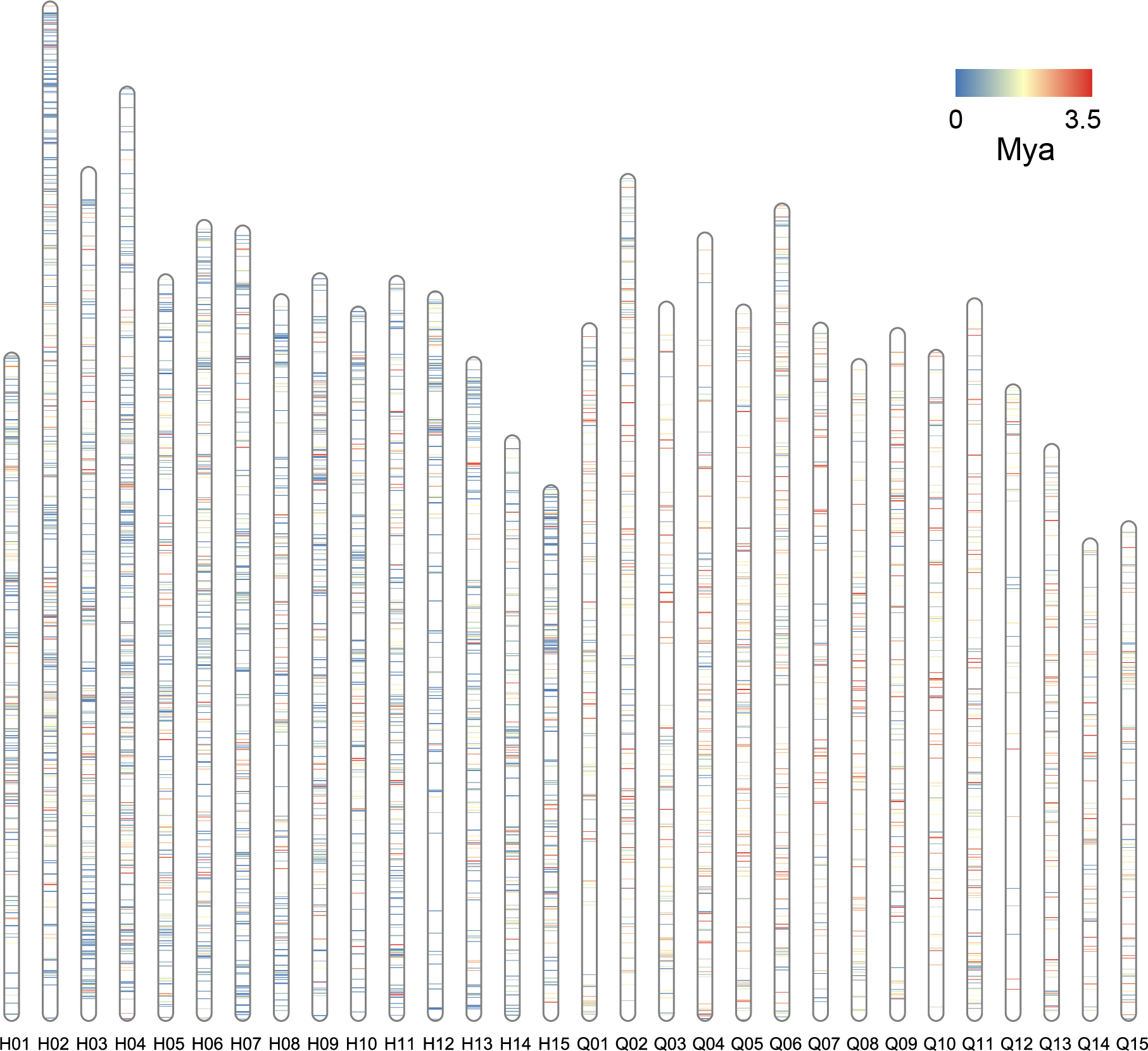


**Supplementary Figure 6.** Density distribution of long terminal repeat (LTR) insertion times and their respective chromosomal positions in *R. punjabensis* (HFY) and *R. potaninii* (QFY).


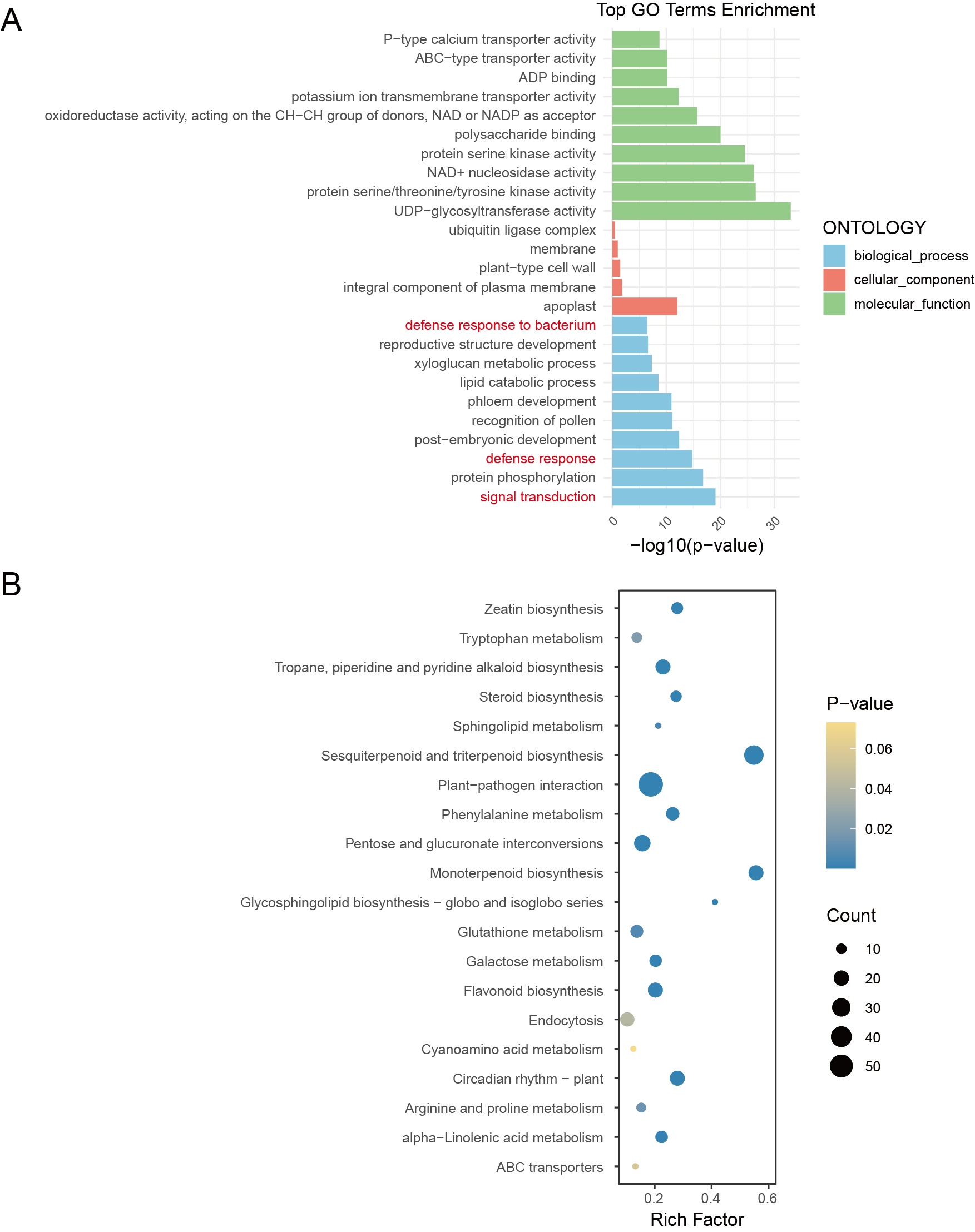


**Supplementary Figure 7.** **Functional enrichment analysis of expanded gene families in Anacardiaceae. (A)** Gene Ontology (GO) enrichment and (**B**) Kyoto Encyclopedia of Genes and Genomes (KEGG) pathway enrichment.


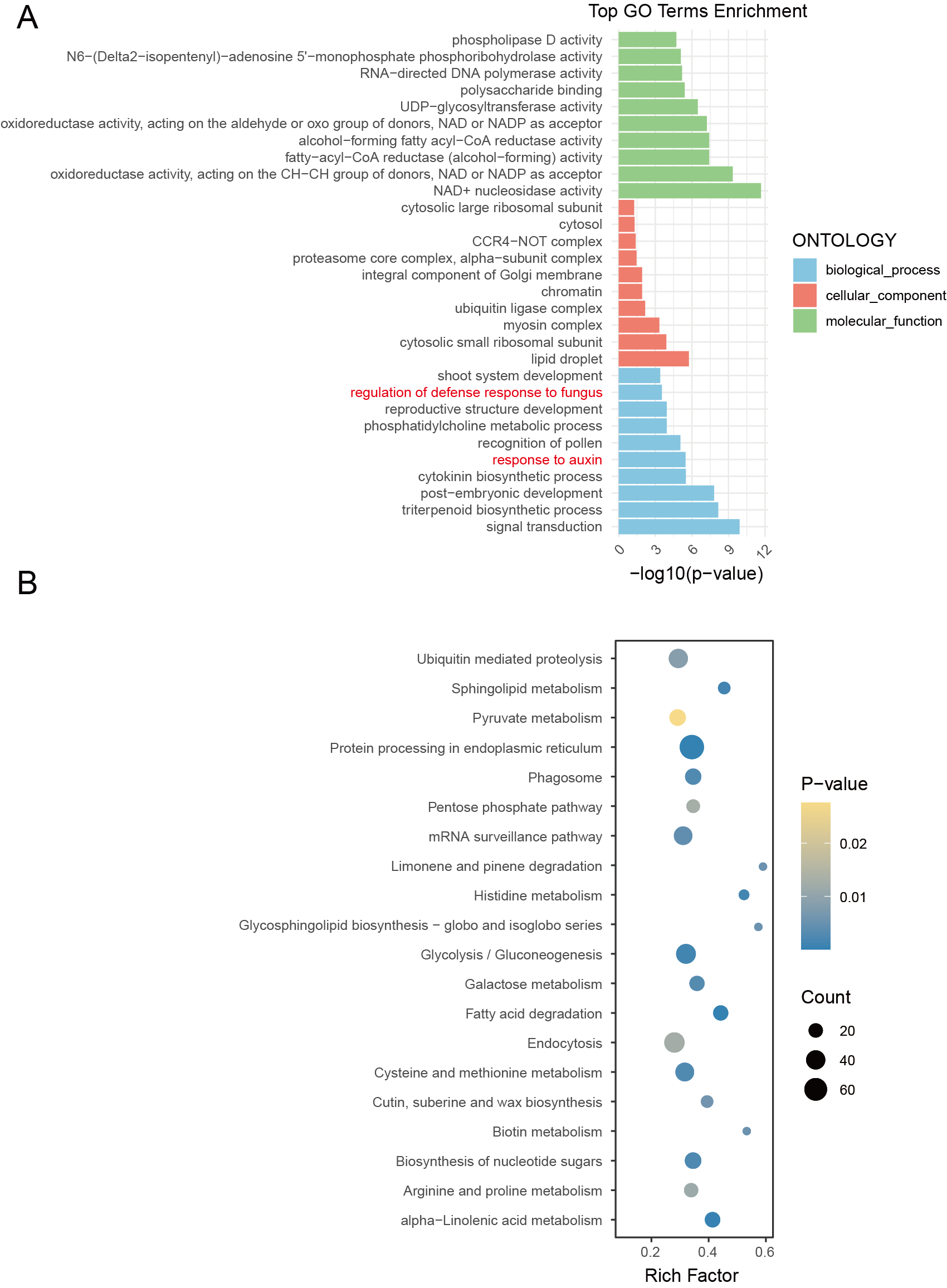


**Supplementary Figure 8.** **Functional enrichment analysis of expanded gene families in *M. indica*. (A)** Gene Ontology (GO) enrichment and (**B**) Kyoto Encyclopedia of Genes and Genomes (KEGG) pathway enrichment.


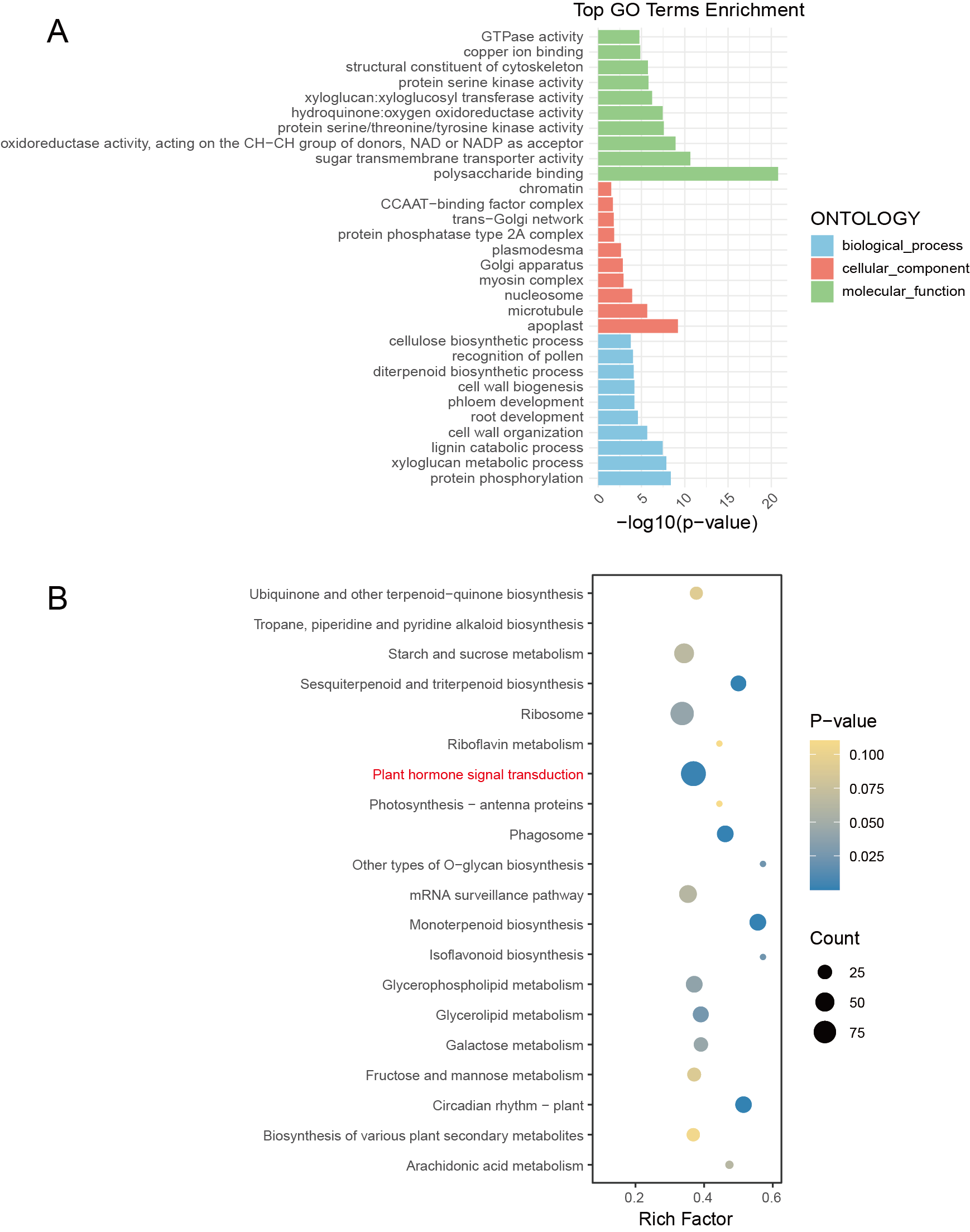


**Supplementary Figure 9.** **Functional enrichment analysis of expanded gene families in** ***A. occidentale*. (A)** Gene Ontology (GO) enrichment and (**B**) Kyoto Encyclopedia of Genes and Genomes (KEGG) pathway enrichment.

**
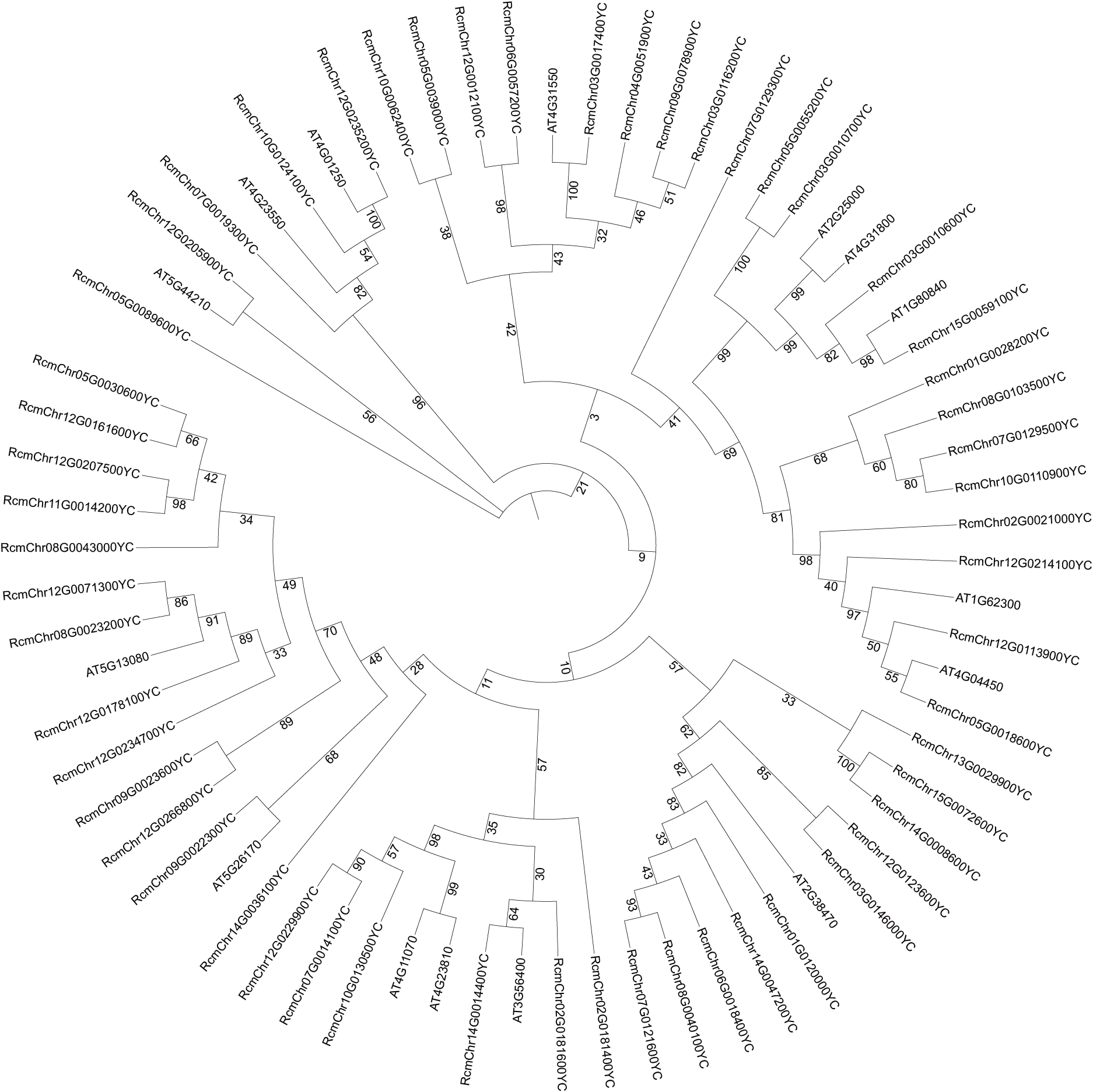
**

**Supplementary Figure 10.** Phylogenetic analysis of 15 representative WRKY domain sequences (PF03106) from *A. thaliana* and 55 WRKY homologs from *R. chinensis*.


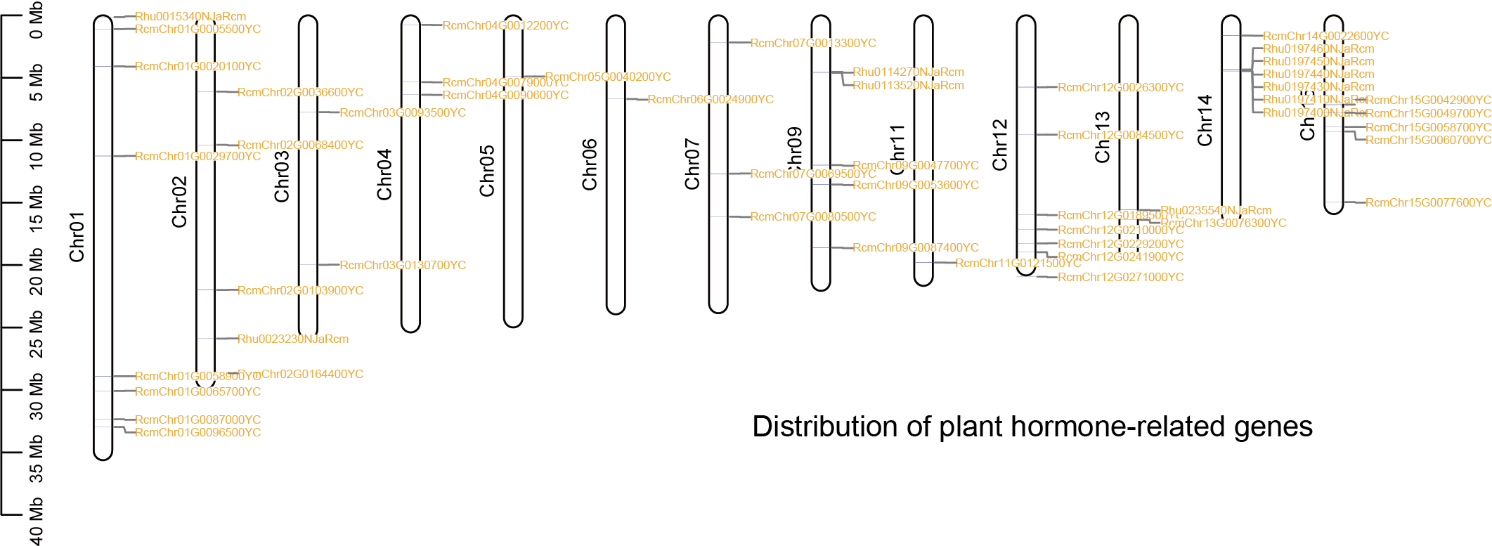


**Supplementary Figure 11.** Chromosomal distribution of phytohormone-associated genes in *R. chinensis*.


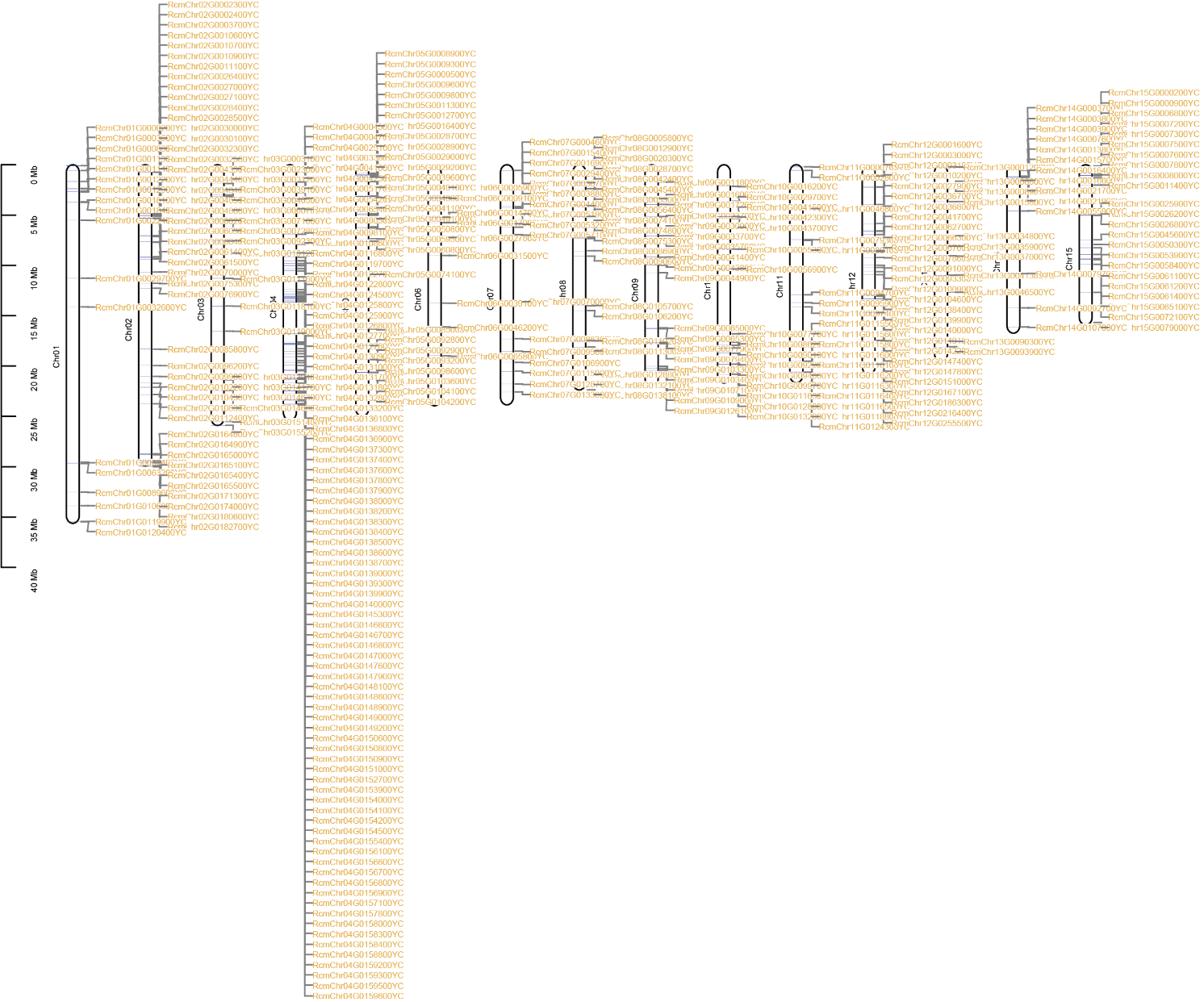


**Supplementary Figure 12.** Chromosomal distribution of nucleotide-binding site leucine-rich repeat (NLR) related genes in *R. chinensis*.


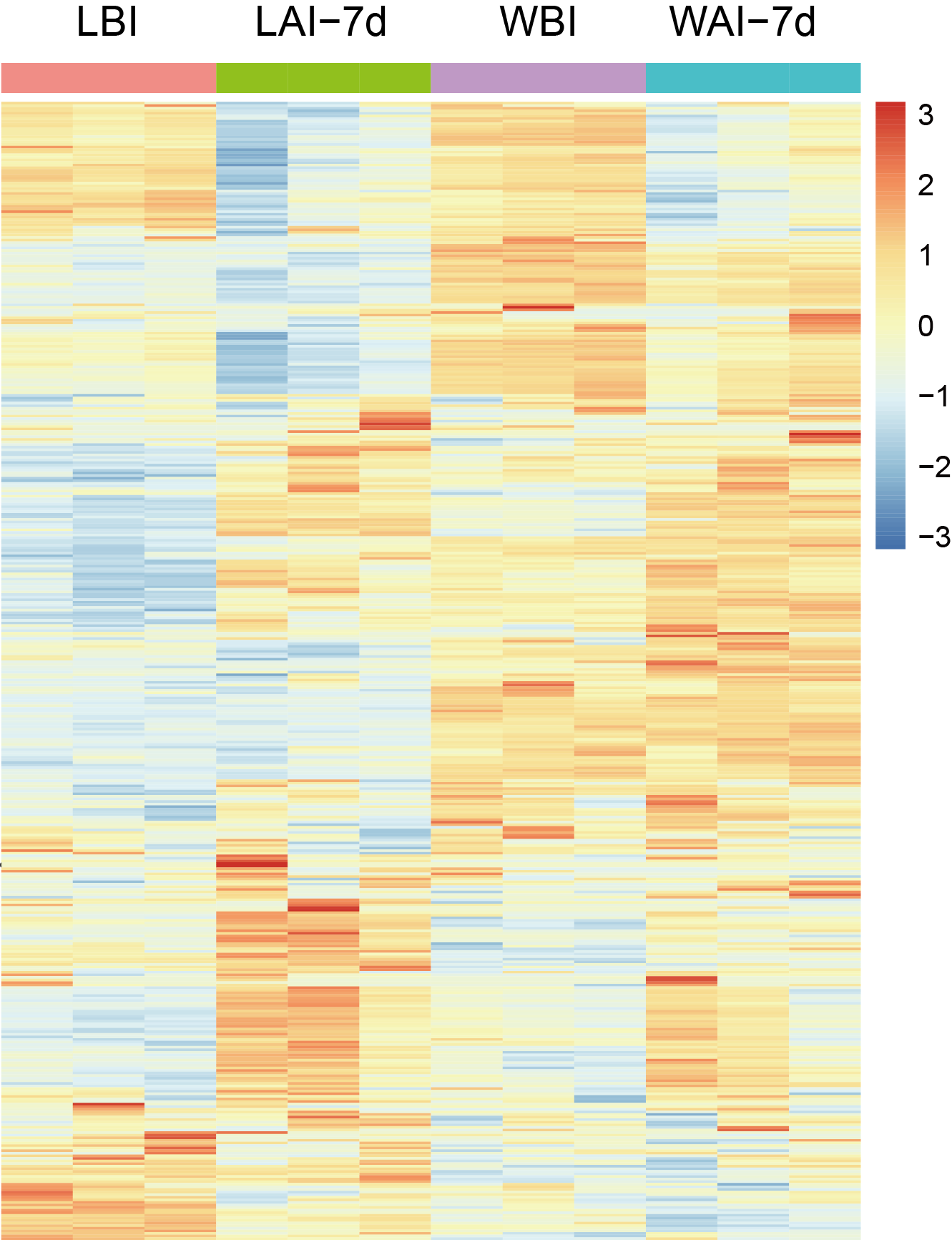


**Supplementary Figure 13.** Expression levels of defense-related genes in four conditions: leaf-wing before infection (WBI), leaf-wing after 7 days of infection (WAI-7d), leaf before infection (LBI), and leaf after 7 days of infection (LAI-7d).
